# Survey on Exact kNN Queries over High-Dimensional Data Space

**DOI:** 10.3390/s23020629

**Published:** 2023-01-05

**Authors:** Nimish Ukey, Zhengyi Yang, Binghao Li, Guangjian Zhang, Yiheng Hu, Wenjie Zhang

**Affiliations:** 1 School of Computer Science and Engineering, University of New South Wales, Sydney, NSW 2052, Australia; 2 School of Minerals and Energy Resources, University of New South Wales, Sydney, NSW 2052, Australia

**Keywords:** kNN queries, kNN Join, kNN Search, high-dimensional data

## Abstract

*k* nearest neighbours (kNN) queries are fundamental in many applications, ranging from data mining, recommendation system and Internet of Things, to Industry 4.0 framework applications. In mining, specifically, it can be used for the classification of human activities, iterative closest point registration and pattern recognition and has also been helpful for intrusion detection systems and fault detection. Due to the importance of kNN queries, many algorithms have been proposed in the literature, for both static and dynamic data. In this paper, we focus on exact kNN queries and present a comprehensive survey of exact kNN queries. In particular, we study two fundamental types of exact kNN queries: the kNN Search queries and the kNN Join queries. Our survey focuses on exact approaches over high-dimensional data space, which covers 20 kNN Search methods and 9 kNN Join methods. To the best of our knowledge, this is the first work of a comprehensive survey of exact kNN queries over high-dimensional datasets. We specifically categorise the algorithms based on indexing strategies, data and space partitioning strategies, clustering techniques and the computing paradigm. We provide useful insights for the evolution of approaches based on the various categorisation factors, as well as the possibility of further expansion. Lastly, we discuss some open challenges and future research directions.

## 1. Introduction

*k* nearest neighbours (kNN) queries are important in many domains, such as data mining, recommendation systems, the Internet of Things (IoT) and the Industry 4.0 framework. We summarise the kNN applications of each domain in Table 1.

We also discuss these applications in Section 6 in detail. In this paper, we focus on two fundamental types of kNN queries, which are kNN Search and kNN Join. In kNN Search, for a given query point, we find its *k* closest neighbours. On the other hand, in kNN Join, we find the *k* nearest neighbour for *all* query points.

We have observed that most of the research has prominently focused on approximation techniques over high-dimensional data. The purpose of the approximate nearest neighbour (ANN) algorithms [1,2,3,4,5] is to improve search efficiency at the cost of accuracy, i.e., a trade-off between search efficiency and accuracy. It means that the resultant nearest neighbours may not really be the true *k* nearest neighbour outcome. On the other hand, the exact kNN approach aims to provide true solutions without compromising accuracy. Since getting the accurate kNN is crucial in many cases of kNN applications, we focus on *exact* kNN query methods in this paper.

**Table 1:** Applications of kNN queries.

Domains	Applications
Sensor Networks	intrusion detection systems [6,7], fault detection [8], fault identification [9], fault classification [10], fall prediction [11], indoor localisation [12,13], etc.
Robotics	arm movement recognition [14], human emotion classification [15,16], scan matching [17], object recognition [18], fast point cloud registration [19], etc.
Mining Industry	predict blast-induced ground vibration in open-pit coal mines [20], safety risk assessment and risk prediction in underground coal mines [21], classification of human activities [22], etc.
Recommendation Systems	recommending products, recommending media to users and showing targeted relevant advertisements to customers and many more [23,24,25]
Data Mining	pattern recognition [26,27,28,29], regression [30,31,32], outlier detection [33,34,35,36]
Machine Learning	text categorisation [37,38], question answering [39], text mining [40], face recognition [41,42], emotion recognition [43,44], image recognition [45,46], handwriting recognition [47,48] and credit card fraud detection [49]
Others	time series [50], economic forecasting and many more applications [51]

**kNN in Low-dimensional Space.** Due to the large number of applications that we discussed in Section 6, kNN queries have been extensively studied in the literature, starting from low-dimensional space. In the early seventies, Donald E. Knuth, referred to this problem as the post-office problem [52], i.e., the location of a house with respect to the closest post office. To find the *k* closest neighbour for any given query point, we can utilise the fundamental kNN Search approach, which is often called the brute force (BF) method or the exhaustive search approach. For the given query points, it scans the entire dataset to find the *k* closest points based on the distances between the query point and all other data points, which is computationally intensive. The cost of the Euclidean distance of a single kNN query is O(nd), where *n* is the number of samples and *d* is the dimensionality of the datasets. When the object dataset is large or when many queries need to be addressed, the query run time increases to a very high level.

The kNN Join was first introduced by Böhm and Krebs [53,54]. The study on kNN Join was inspired by the fact that computing the nearest neighbour for all query points at once speeds up the performance significantly compared to computing individually. It helps to improve the performance of many applications, such as k means clustering [55,56], outlier detection [57,58], kNN classification [59,60], k distance diagrams, missing value computation, etc. [54].

Several studies address kNN queries in low-dimensional space [61,62,63,64,65,66,67,68]. In order to efficiently process the low-dimensional datasets, a variety of R-tree versions with different heuristic improvements have been proposed. R-tree [61] divides the minimum bounding rectangle (MBR) and when dividing an MBR, R*-tree [62] takes overlap into account. This helps to enhance search efficiency. In the Hilbert R-tree [63], related MBRs are grouped together based on Hilbert ordering. In PR-trees [64], priority rectangles are used to handle large volumes of data. These variations of the R-tree are primarily used to index low-dimensional data. In addition, K-Dimensional tree (KD-tree) [65] is a popular approach for avoiding exhaustively comparing the query point with every data item point. It divides the feature space into a binary search tree, which is used to quickly find the closest neighbours of any point. The branch and bound approach (Ball-tree) [66] was employed to provide the speedy computation of the kNN by removing the requirement to calculate several distances. As a more effective approach to these challenging search issues, the vantage point tree (VP tree) [67] is offered in different variations. Both KD trees and VP trees can be seen as very exceptional examples belonging to the divide-and-conquer algorithmic paradigm and deriving from certain uniformly continuous functionals. In [68], the researchers provide the multi-vantage point (MVP) tree, a distance-based index structure for similarity searches. The MVP tree divides the space into spherical slices at each level using multiple vantage points. It also makes use of the pre-calculated distances between the data points and the vantage points.

However, these effective low-dimensional algorithms are unable to scale well when dealing with high-dimensional (HD) datasets.

**kNN in High-dimensional Space.** Several application fields, such as e-commerce, network security, molecular biology, industrial applications and many more, have become more important in recent years and represent their data as high-dimensional feature vectors. For example, the massive enhancement of products and users in recent years has encountered several significant issues. The recommendation system [23,24,25] has been used in many different fields and it helps to find product suggestions on e-commerce sites while users are actively using the site. Moreover, network security and network protection are becoming more important than ever before because of the exponential rise of network-based services and the sharing of information on networks, which also raises the threat of network attacks and breaches [69,70]. The key research problem in these fields is the object-based searching of relevant multimedia objects such as images, videos, audio, etc.

Certain traditional indexing algorithms, such as the B-tree and R-tree families, are optimised for a small number of dimensions. However, as the number of dimensions grows, the performance of these algorithms degrades faster. As a result, a sequential linear scan emerges as the quickest retrieval technique. This problem is often called the “curse of dimensionality” [71,72,73]. The typical strategy to deal with this problem is to either extend these low-dimensional kNN query techniques or propose new approaches. Therefore, during the last several years, there have been a lot of studies done on kNN query processing in high-dimensional spaces and many novel high-dimensional approaches [74,75,76] have been presented. Processing HD datasets is challenging. Even if we consider moderate-sized datasets, high dimensionality can act as an additional potential problem for them. Moreover, the combination of HD datasets and large-sized datasets in the real world can pose additional difficulty. So, in order to use the HD dataset, it needs to be processed with the help of efficient HD techniques because, as we discussed earlier, it is difficult for traditional low-dimensional approaches to be effective in high-dimensional space.

The kNN approach encounters two common challenges in high-dimensional space: finding the kNN and computing distance. To speed up the kNN queries, many solutions are available with datasets of different sizes, dimensionalities and distributions. Below, we mentioned some commonly used strategies.

*Parallelisation.* One solution to improve the performance is to parallelise the computation, which can be done using a cluster, GPU or multiple cores on a single machine. Rather than reducing the number of computations, this method divides the task into many parts, which are processed concurrently on separate processing units. For instance, the researchers in [77] show a significant speedup by parallelising the brute-force search using GPUs.

*Dimensionality reduction.* Dimensionality reduction (DR) techniques map the data points from an HD space to a 1D or low-dimensional space because searching in a lower-dimensional space is faster and more cost-effective. Various approaches have been proposed to reduce dimensionality. For example, Principal component analysis (PCA) [78,79,80,81] is one of the most famous and widely used Dimensionality Reduction Approaches because of its efficiency and scalability.

*Partitioning methods.* The goal of the approaches that follow the partitioning strategies is to minimise the distance computation and speed up the search. We classified all the approaches into two broad categories, i.e., *space-based* and *data-based*. Details can be found in Section 3. The data points are used to create a tree structure that divides the data space. The resultant tree effectively prunes the unnecessary distance calculation. There are several existing techniques that use various tree structures, such as R-tree, R*-tree, Ball tree, KD-tree, etc. These approaches perform well for low-dimensional data but usually do not work well for high-dimensional datasets, as mentioned before. Therefore, different new tree structures have been proposed to deal with high-dimensional datasets such as M-tree [82], Δ-tree [74], HDR-tree [83], etc.

**Survey Scope.** Our survey in this paper focuses on exact kNN querying techniques (i.e., kNN Search and kNN Join) over high-dimensional space. Given that an increasing number of works have addressed exact kNN queries over high-dimensional data in recent years, the existing survey only studies kNN in low-dimensional space [84,85,86] or approximate approaches over high-dimensional data [87,88]. In addition, MapReduce-based (distributed and parallel) survey work is presented in [89,90] but there is no work available that focuses on all the exact kNN Join techniques. We aim to provide a comprehensive overview of existing techniques along with their classification and a comparative analysis, which is proposed for HD datasets. We summarise the motivation of our work in the following.

1. kNN queries in high-dimensional space are widely used and are becoming increasingly popular in various applications in recent years.
2. In kNN Search, though many works have been proposed in the literature, there is no survey on exact kNN over high-dimensional data.
3. In kNN Join, the comparative work of various exact and approximate MapReduce-based approaches is studied [89,90], but there is no survey available that prominently focuses on all the exact kNN Join techniques over high-dimensional space.

**Contribution.** This paper outlines a detailed analysis of the state-of-the-art solutions for kNN Search as well as kNN Join over high-dimensional data. Following are our contributions:

1. We present a comprehensive overview of the kNN queries over high-dimensional data, which covers 20 kNN Search methods and 9 kNN Join methods. As per our knowledge, this is the first detailed study of the exact kNN approaches in high-dimensional data space.
2. We systematically classify and compare existing strategies. For each approach, we explain in detail its method, basic features, as well as its strengths and weaknesses over other methods. As a result, we summed up the existing techniques.
3. We discuss a number of open challenges as well as future research directions for resolving kNN query problems.

**Outline of the Paper.** Further sections of the paper are structured as follows. We define the basic terminology along with the problem definition in Section 2. In Section 3, we classify various kNN queries considering different classification factors. In Section 4 and Section 5, we give an overview of the kNN queries and discuss all the kNN Search and kNN Join approaches based on the Computing Paradigm classification strategy. Applications of kNN are discussed in Section 6. In Section 7, we provide a comparative study of the discussed approaches and a conclusion in Section 8. Finally, challenges and future directions are discussed in Section 9.

## 2. Background

In this section, we give formal definitions of the most common terms used in this paper and also discuss related work. A summary of frequently used symbols is given in Table 2.

### 2.1. Definitions

Here, we provide the definitions for kNN Search, kNN Join, distance range, k Distance Join and the reverse kNN Join operation.

**Definition** **1 (kNN Search).** *Let $R=\{r_{1},r_{2},\ldots ,r_{i}\}$ be a set of data points in d-dimensional space $R^{d}$, q be a query data point in $R^{d}$, the function $d(q,r_{i})$ to compute the distance between two data points q, and $r_{i}$ be the distance function and k be a positive natural number. Then, the result of the kNN Search with respect to q and R is an ordered collection, kNN(R,q,k)⊆R, which contains k(1≤k≤|R|) different data points with the k least distances from q, such that $kNN\left(R,q,k\right)=\left\{r_{1},r_{2},\ldots ,r_{k}\right\}\subseteq R$, $d\left(r_{i},q\right)\leq d\left(r_{j},q\right)$ if 1≤i<j≤k and ∀r∈R|kNN(R,q,K); we have $d\left(r_{i},q\right)\leq d\left(r,q\right),1\leq i\leq k$.*

**Figure 1:**
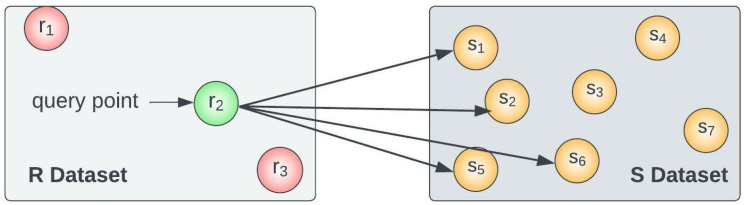
An example of kNN Search with *k* = 4.

**Example** **1**. *Figure 1 depicts an example of a kNN Search with k = 4. For the query point of dataset $R=\{r_{1},r_{2},r_{3}\}$, the kNN Search process finds the four nearest neighbours from the object dataset $S=\{s_{1},s_{2},s_{3},\ldots ,s_{7}\}$.*

In order to facilitate similarity searches, the similarity join has emerged as a crucial fundamental database. It combines the two sets of multidimensional/high-dimensional data so that the result is all the pairs of similar objects. The similarity join can be categorised mainly into two types: the distance range which is also called distance range join and the k Distance Join. In a study, Böhm and Krebs [53] introduced the third type of similarity join, i.e., kNN Join. Sometimes, incremental distance join is also considered a type of similarity join.

**Definition** **2 (kNN Join).** *Let R and S be the two datasets of data points in d-dimensional space $R^{d}$, the function $d(r_{i},s_{j})$ to compute the distance between two data points $r_{i}$ and $s_{j}$ be the Euclidean distance function and k be a positive natural number. Then, the result of kNN Join query is a set kNNJ(R,S,k)⊆R×S, which includes for every point of $R(r_{i}\in R)$ its k closest neighbours in $S:kNNJ\left(R,S,k\right)=\{\left(r_{i},s_{j}\right):r_{i}\in R,s_{j}\in kNN\left(S,r_{i},k\right)\}$.*

**Figure 2:**
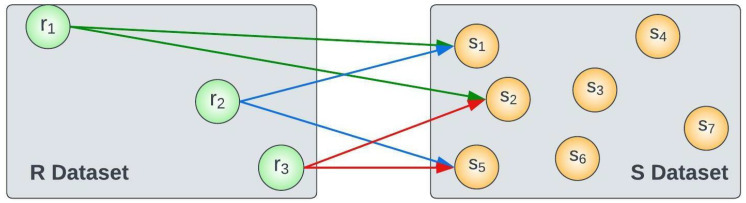
An example of kNN Join with *k* = 2.

**Example** **2**. *As shown in Figure 2, for a given value k = 2, the kNN Join process finds the two nearest neighbours from the object dataset $S=\{s_{1},s_{2},s_{3},\ldots ,s_{7}\}$ for every item in the query dataset $R=\{r_{1},r_{2},r_{3}\}$.*

**Definition** **3 (Distance Range).** *A distance range can be defined as for the given two datasets, i.e., $R=\{r_{1},r_{2},r_{3},\ldots ,r_{n}\}$ and $S=\{s_{1},s_{2},s_{3},\ldots ,s_{m}\}$, finding the set of all pairs within a given distance range that {(r,s):r∈R,s∈S,d(r,s)≤θ}, where θ is a threshold defined by the users.*

The distance range join is the most common and a much-studied similarity join technique. As a result, “similarity join” and “distance range join” are frequently used synonymously.

**Definition** **4 (k-Distance Join).** *The closest point query, also known as the k Distance Join, can be defined as for the given two datasets, i.e., $R=\{r_{1},r_{2},r_{3},\ldots ,r_{n}\}$ and $S=\{s_{1},s_{2},s_{3},\ldots ,s_{m}\}$, finding the set of the k most similar pairs crossing R and S so that KDJ(R,S)⊆R×S, KDJ(R,S) has k elements where 1≤k≤min(n,m) and $d\left(r_{i},s_{j}\right)\leq d\left(r_{k},s_{l}\right)$ if $\left(r_{i},s_{j}\right)\in KDJ\left(R,S\right)$ and $(r_{k},s_{l})\in$R × S|KDJ(R,S).*

**Definition** **5 (Reverse kNN Join).** *Let R and S be the two datasets of points in d-dimensional space $R^{d}$, the Euclidean distance function $d(r_{i},s_{j})$ compute the distance between two data points $r_{i}$ and $s_{j}$ and the natural number $k\in N^{+}$. Then, the results of reverse kNN Join with respect to the query data point $s_{j}$ is a set of data points $RkNN(R,s_{j})\subseteq R$ that includes $s_{j}$ as one of their kNNs. $RkNN\left(R,s_{j},k\right)=\left\{r_{1},r_{2},\ldots ,r_{n}\right\}\subseteq R$, such that $\forall r\in R\wedge s_{j}\in S$.*

**Definition** **6 (Dynamic kNN Join).** *Let R and S be the two datasets of points in d-dimensional space $R^{d}$, the Euclidean distance function $d(r_{i},s_{j})$ compute the distance between two data points $r_{i}$ and $s_{j}$ and the natural number $k\in N^{+}$. Then, the dynamic kNN Join is the ability to dynamically join similar data points DkNNJ(R,S,k)⊆R × S in $R^{d}$, which includes for every point of R its k(1≤k≤|S|) closest neighbours in $S:DkNNJ\left(R,S,k\right)=\left(r_{i},s_{j}\right):\forall r_{i}\in R,s_{j}\in kNN\left(S,r_{i},k\right)$ and maintains (updates) the complete join result with every update operation, i.e., for insertion or deletion of any data item $s_{i}\in S$, finding the affected user set $r_{a}:RkNN\left(s_{i}\right)|s_{i}\in S\wedge RkNN\left(s_{i}\right)\subset R$ and updating the affected user set $kNN(S,r_{a},k)\subseteq R$ × S.*

### 2.2. Related Work

It is a prevalent belief that finding the exact kNN over a high-dimensional dataset is a very expensive operation. The approximate nearest neighbour search (ANNS) does not guarantee providing the true nearest neighbours (NNs), but it sufficiently returns the nearby data points. It can be carried out effectively and is adequate for a wide range of applications, attracting a great deal of research effort. 

**Approximate Nearest Neighbour.** Let $S=\{s_{1},s_{2},\ldots ,s_{i}\}$ be a set of data points in *d*-dimensional space $R^{d}$, *q* be a query data point in $R^{d}$ and $s^{*}$ be the *q*’s kNN $r^{*}=d\left(q,s^{*}\right)$. The *s* is the *k*-th nearest neighbour of *q* and $r^{p}=d\left(q,p\right)$. Given θ>0 (or c>1), then $\left(s,r^{p}\right)\in R^{d}$ is a (1+θ)-approximate (or *c*-approximate) solution to the kNN query kNN(q,S) if $r^{*}\leq r^{p}\leq \left(1+\theta \right)r^{*}$ (or $r^{*}\leq r^{p}\leq cr^{*}$ for some constant *c*). 

In several works, the issue of ANNS over HD data has been widely studied. Hundreds of works have been proposed in order to address the issues from various perspectives. The curse of dimensionality causes researchers to focus on the approximate solution, as it returns as many true nearest neighbours as possible. In a few surveys, researchers talked about the latest ANNS techniques and gave a full experimental analysis. The cost of a brute-force kNN Search is O(d.n). Although we can overcome the linear dependency on *n*, the high dimensionality of the dataset remains a challenge. Here, *n* is the number of data points and *d* is the dimensionality of the dataset. In some cases, it is not necessary to search for the exact closest neighbours. For example, in recommendation systems, the most similar item is not always needed; in fact, things that are moderately relevant may provide some possibility for unexpected discovery. In the kNN classification, neighbours that are near one another but not essentially the closest ones are likely to belong to the same class.

ANN algorithms provide faster results over a high-dimensional dataset. These algorithms are mostly classified into three main categories: hashing-based, partition-based and graph-based. In ANN, locality-sensitive hashing (LSH)-based techniques [1] are very famous. Here, the goal is to apply specific hash functions to effectively map data points to discrete buckets, ensuring that points with similar characteristics are placed in the same bucket.

**k-Nearest Neighbour Graph.** The kNN graph (kNNG) for a set of data points (vertices) *V* has an edge connecting each v∈V to its corresponding kNN in *V*. The graph-based techniques constructed a proximity graph in which each data point in the dataset $R=\{r_{1},r_{2},\ldots ,r_{i}\}$ is represented as a node and the edges connecting some nodes to their kNN form a kNNG G(V,E) in *d*-dimensional space $R^{d}$. These approaches are based on the principle that a neighbour’s neighbour is probably also a neighbour. Its building process is a significant one that has a lot of applications on the web, such as collaborative filtering [91], similarity search and many more in data mining and machine learning [92]. Nearest neighbours are retrieved for the given query point using greedy, implementation-specific graph traversals [93,94,95]. The brute-force approach to kNNG construction is only feasible for small datasets and has an $O(n^{2})$ cost. The classifications for graph-based approaches are broad. One can build an exact or approximate kNNG that records the top-k NN for every node. Approximation kNNG generation techniques have recently received a lot of attention, especially in high-dimensional space [96,97,98,99,100]. 

The issue of high dimensionality and large datasets in graph-based ANNS has been extensively researched in the literature [101]. To solve this problem, many methods have been proposed using different optimisation techniques [95,102,103,104,105]. Existing surveys [88,106,107,108] carried out comprehensive comparative studies, conducted experimental analyses of the existing approaches and provided some useful insights.

## 3. Classification of kNN Queries

Our work categorises exact kNN query algorithms based on five factors, offering a different perspective on existing work. These factors enable us to group similar approaches together, which provides a basis for further expansion. It is easy to identify the problems when they use similar methods. Therefore, we categorise the existing approaches based on their techniques.

Here, we classified all the *k* nearest neighbour query approaches based on the following factors:

1. Indexing Technique—index-based techniques speed up the kNN Search operation over HD datasets with an extra space cost of building certain data structures in advance. We discussed a few commonly used index structures such as R-trees, iDistance and so on in the below Indexing Technique section.
2. Partitioning strategies—this partitions the whole dataset based on data-based and space-based partitioning. Using an appropriate partitioning strategy determines how well an approach will perform, which makes it an important computational parameter. The *space-based* approach does not require any knowledge of the actual dataset. It divides the entire data space into two or more partitions and is recursively applied to every newly generated region to further partition the space. On the other hand, a *data-based* approach adjusts the size and position of divisions based on the distribution of the data.
3. Dimensionality reduction strategy—this is computationally intensive in terms of processing the HD dataset. Thus, various researchers used the DR strategy, which helps in reducing the dimensions in order to process them efficiently. It basically reduces the dimensionality by projecting the high-dimensional data to a low-dimensional space, which captures the majority of important information. Examples include PCA, iDistance, etc.
4. Distance computation approach—when determining the kNN, the Distance Metric plays a very important role. So, to search for the k closest neighbour data points, we need to find the distance between the query point and all the other data points. Many computation approaches are available to find the similarity between points, but in our case, the majority of approaches use the *Euclidean distance*.
5. Computing Paradigm—we also discuss (in Section 4 and Section 5) the various computing approaches, such as memory-based, I/O-based, parallel and distributed.

**Figure 3:**
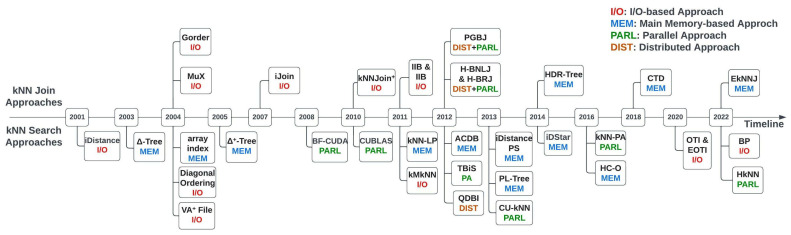
Timeline of kNN Join and kNN Searching techniques.

**Figure 4:**
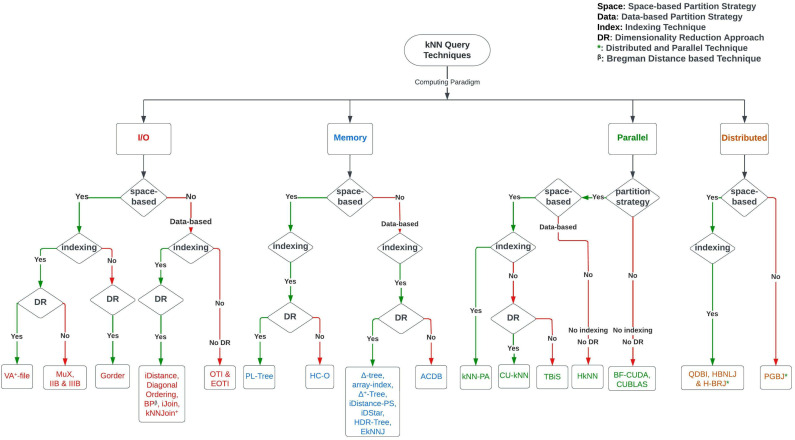
Block flow diagram of kNN query techniques based on the different classification categories.

For a clear understanding of the methods and their development, we have included a timeline of the kNN Join and kNN Search techniques in Figure 3. We included all of the kNN Join techniques in the top region of the figure and the kNN Search techniques in the lower region. In Figure 4, we also present the block flow diagram of exact kNN query techniques based on the different classification categories we talked about above. Almost all the kNN queries that we have discussed in our work utilise Euclidean distance, except the BP technique, which uses the Bregman distance. Here, we indicate the Bregman distance-based technique with the β symbol. The * symbol denotes a method that employs both distributed and parallel Computing Paradigms. 

In Table 3 and Table 4, we have provided a comparative study of existing kNN Search and kNN Join techniques. We compared the techniques based on different classification strategies such as the Indexing Technique, Partitioning Approach, Dimensionality Reduction Approach, Computing Paradigm and also Distance Metric. We also provided additional information such as the approach used, i.e., exact or both (exact+approximate), dataset dimensionality and whether the researchers took dynamic datasets into account or not. Here, we tried to summarise all the existing techniques, and details about them are discussed in Section 4 and Section 5 to get a gist of their approach.

In Table 3 and Table 4, we used several abbreviations. We used “Both” to indicate that the work provided both solutions, i.e., exact and approximate. Many researchers have discussed the approximate strategy to improve the effectiveness of an exact technique in their work. In this paper, we focus only on the exact solutions. So, readers can refer to their work if they want to dig deep into it. Dimensionality is denoted as “Dim.” in the table. We have indicated the three different ranges here—mod., high and high+—which are termed moderate high-dimensional datasets, high-dimensional datasets and very high-dimensional datasets. There is not any standard dimension for high dimensionality. For example, work with 12D [117], 30D [112], 32D [109] and 500D [129] datasets is regarded as a high-dimensional dataset. Therefore, considering the scenario in mind, we divide the dimensionality into three different ranges. Most existing techniques are classified into the first category. We included all 2D to 99D in the first range. We included works in the second range that conducted experiments on datasets with dimensionalities ranging from 100D to 499D. In the third range, we consider the dataset to have a dimensionality equal to or greater than 500D. “Dyn. Data” is referred to as whether the existing approach uses dynamic datasets or not. Time complexity is denoted as “Time comp.” The majority of the techniques did not include an algorithm with time complexity in their work. Therefore, we calculated it based on the related papers’ descriptions and experimental estimates. We marked them with * (asterisk) symbols. We basically computed the kNN for the *n* data points. 

In iDistance [110,111] and iDistance-PS [120], the researchers tested both Partitioning Approaches, namely space-based and data-based approaches. To avoid the complexity of tables, we mention them as “data-based” in the partitioning strategy taxonomy, as it was shown in their experimental study that the data-based approach performed better than the space-based approach. On the other hand, in the kNN-PA [123] work, they also performed the experiments using both the strategies, i.e., cluster-based (data-based) and hyperplane-based (space-based). They observed that the space-based approach outperforms the data-based approach. Therefore, we categorise this approach as the space-based approach.

As shown in Table 3 and Table 4, some of the existing techniques use traditional indexing strategies (like the R-tree and B-tree families). The majority of the approaches propose their own indexing strategies or use various different kinds of Indexing Techniques. It can be seen that some parallel and distributed approaches do not implement any indexing strategy, such as BF-CUDA [77,131], CUBLAS [75], CU-kNN [121], TBiS [122], etc. Thus, for those who are not using any Indexing Techniques, we mark them as “N/A” in the table. We classified the Partitioning Approaches into two major categories, i.e., Data (data-based) and Space (space-based) Partitioning. Here, we consider the cluster-based partitioning strategy as a data-based technique. We add all the remaining approaches that do not use any partition strategy into the “No Partition” category. In the dimensionality-reduction approach, the majority of the parallel and distributed approaches do not use dimensionality-reduction techniques. Therefore, we mark them as N/A. The rest use either the PCA, iDistance or a different reduction strategy. The shortcuts used in the Computing Paradigm stand for I/O-based (I/O), Memory-based (Memory), Parallel and Distributed. In the Computing Paradigm, we specified the predominant approach for a given technique. For example, if any technique is mentioned as an I/O-based technique, that does not mean it is an I/O-based technique only. It also involves main memory, but since the significance of I/O was much greater as compared to main memory, we classified it as an I/O technique. Memory-based approaches are those in which the majority of the operations are carried out in the main memory. For the Distance Metric, almost all approaches use Euclidean distance except the BrePartition (BP) Approach. It uses non-metric Bregman divergences for distance computation.

### 3.1. Indexing Technique

Index-based techniques seem to be a more promising solution because they have the ability to perform a faster kNN Search operation over a high-dimensional dataset. Many researchers worked on the nearest neighbour query problem in high-dimensional space and came up with novel indexing strategies such as R-trees [61], B${}^{+}$-tree [132], pyramid technique [133], M-Tree [82], iDistance [110,111], etc., to solve the problems with existing methods for finding kNN over high-dimensional data.

The index-based kNN queries involve significant I/O overhead due to the high number of accesses, but they are optimised for CPU cost. Furthermore, the index often fails in high-dimensional space, where it performs worse than a sequential scan. Many researchers have come up with novel, efficient solutions to address this issue.

As mentioned in Table 3 and Table 4, some existing techniques use traditional indexing strategies (such as the R-tree and B-tree families). On the other hand, the majority of the approaches use various different kinds of Indexing Techniques. Like the array-index approach [117,134], it uses a one-dimensional array-index, which is a simple, compact, but still efficient index structure. Researchers present a new indexing strategy known as a Δ-tree [74] to accelerate the processing of high-dimensional kNN queries in main-memory. It is a multi-tiered structure where every level represents a different dimension. These multi-level structures provide better pruning power and lower distance computation costs. To make the search power more effective, they also proposed the $\Delta^{+}$-tree approach [74,118]. For high-dimensional indexing, the kNN query process is enhanced by using adaptive cluster distance bounds in the study of [119]. For effective data management and query processing in peer-to-peer systems, the researchers came up with a distributed multidimensional data index (QDBI) [125] that is based on quad-trees. Here, every peer uses an MX-CIF quad-tree to generate index items for their high-dimensional data. Every index item then obtains a code in accordance with the MX-CIF quad-tree. To enable effective point queries, range queries and kNN queries, the authors introduce a novel Indexing Technique, PL-Tree [115]. This method uses algebraic methods to dynamically index items, which helps to deal with the problem of high dimensionality. The HC-O [116] technique uses a hash-based index. It is a global approach, which works with both accurate tree-based indexes and LSH methods. The hash-based index stores point identifiers in its hash buckets. To speed up search performance, they created an effective integrated index structure [114] that includes all of the subspaces and uses Bregman Ball trees (BB-trees) in partitioned low-dimensional subspaces. This structure is called the BB-forest. BB-trees [135] are effective at handling low-dimensional data; they are a good fit for the approach where data is divided into low-dimensional subspaces. It can be seen that some parallel and distributed kNN query approaches do not implement any indexing strategy such as BF-CUDA [77,131], CUBLAS [75], CU-kNN [121], TBiS [122], etc. Therefore, we indicated all these techniques as “N/A” in the tables.

**R-tree family.** One of the most common index structures is the R-tree family, which includes several multidimensional and high-dimensional indexing techniques [61,62,63,64,136,137,138]. A dynamic, balanced indexing structure called an R-tree uses MBRs to describe data division. If an R-tree node already has enough MBRs, it splits into two nodes. It can be observed that the different R-tree versions use a varied Partitioning Approach with various heuristic improvements. R-trees [61] partition the MBR, and R*-trees [62] take into account overlap when dividing an MBR, which results in enhanced search efficiency. The Hilbert R-tree [63] is used to group together related MBRs with the help of an ordering based on the Hilbert curve. For high volumes of data, PR-trees [64] employ priority rectangles. However, all these variations of the R-tree are mostly used for indexing low-dimensional data and for managing high-dimensional data, a handful of R-tree-based structures such as TV-trees and X-trees are devised. TV-trees [137] minimise dimensionality by storing just the most crucial information about data items, i.e., by arranging dimensions according to their significance. X-trees [136] propose the idea of supernodes to reduce the overlapping in high-dimensional space that keeps the directory as hierarchical as possible and to try to prevent division in the directory. Yang, C. et al. [83] present a novel index structure called the HDR-Tree to efficiently find affected users. It performs dimensionality reduction by using clustering and PCA to improve search effectiveness. A detailed discussion of the HDR-Tree is available in Section 5.

**B${}^{+}$-tree.** The objective of a few modern studies was to design Indexing Techniques that could provide a one-way lossy mapping function from a multi-dimensional/high-dimensional space to a one-dimensional space, which could be effectively indexed in a conventional B${}^{+}$-tree. The use of the B${}^{+}$-tree structure is helpful for the method because it incorporates all of its properties, such as quick search, dynamic updating and height-balanced structure. Additionally, it is simple to use the B${}^{+}$-tree method on any existing approaches.

**iDistance.** The iDistance divides the data space into *m* parts, assigns *m* reference points to each partition and then converts each partition into a one-dimensional space based on the similarity of other data points to the reference point. These values are then mapped to the B${}^{+}$-tree so that they can be searched, accessed and updated more quickly. We have discussed the iDistance indexing strategy in Section 1. You can also refer to the original work of iDistance [110,111] for more detailed info.

**Others.** In [109], the authors introduce an approach based on the scalar quantisation of data called the VA${}^{+}$-file approach. It is mostly useful for searching the kNN in non-uniform datasets. In kNN-PA [123], researchers introduced the parallel tree-building technique called randomised k dimensional tree for indexing structures. This method supports different kinds of trees, such as ball trees, KD-trees, etc., and is used to partition and filter spatial searches. The inverted index approach [127] uses inverted lists. It helps to avoid unnecessary traversals to every item in the object dataset. They are more advantageous for the sparse dataset.

In the kNN Join Indexing Technique category, the iDistance indexing strategy (B${}^{+}$-tree based index structure) is used in a few techniques. iJoin and kNNJoin${}^{+}$ both use the iDistance approach; we renamed it iDistance rather than B${}^{+}$ tree to make it easier for readers to understand. The term “N/A” refers to techniques that do not employ any indexing strategy.

### 3.2. Partitioning Strategies

In this section, we have classified various high-dimensional kNN query techniques based on the partitioning schemes. As shown in Table 3 and Table 4, we basically divided the approaches into three categories, i.e., space-based (Space) partitioning, data-based (Data) partitioning and no partitioning (No Partition). The data-based approaches can be further divided into Voronoi-diagram-based partitioning, cluster-based partitioning and others. However, we do not discuss them in detail to ease our presentation. Most of the approaches adopted the k means clustering approach. Some parallel and distributed approaches did not use any partitioning strategies. So, we added the rest of the approaches to the “Others” category. Approaches like BF-CUDA and CUBLAS, which do not use any partitioning strategy, are added to the “No Partition” category.

**Data-based Partitioning strategy.** A collection of data points is divided into a number of groups using a partitional data-based Partitioning Approach. It creates k(N≥k) divisions of the data, each of which represents a cluster, where *N* indicates the number of data points. In other words, it divides the data into *k* groups by meeting the criteria listed below: Each point belongs to precisely one group and every group includes at least one point. Data Partitioning algorithms work better for creating indexes because of their adaptability to data distributions. For example, the performance of techniques such as Δ-tree [74], $\Delta^{+}$-tree [74,118], BP [114], kNNJoin${}^{+}$ [76], HDR-tree [83], EkNNJ [129], etc., is greatly improved with a data-based partitioning strategy. This makes retrieval much faster in real-world environments. Because many real-world datasets are not homogeneous, data-based partitioning techniques get an advantage. For example, the researchers carried out several experiments on the iDistance technique using different partitioning techniques and they found that the data-based partitioning method of iDistance consistently outperforms the other methods. The main benefit of iDistance is its data-adaptive indexing and it is also shown in their work [110,111] how it helps to enhance the overall performance.

**Space-based partitioning strategy.** Space Partitioning algorithms divide the space into two or more subsets or regions in a fixed way (i.e., uniformly) or in a way that changes over time (i.e., adaptively). It is an effective technique to organise data in *d*-dimensional space. The space-based sort does not require the information of the original data. Tree-type data structures are often related to traditional Space Partitioning. Due to the fact that data distributions for a higher feature space are likely to be non-uniform and sparse, techniques based on regular space partitions can suffer from significant overhead costs [71].

Several approaches use standard partitioning schemes for high-dimensional data spaces. For example, R-tree-based approaches represent the space partition via MBRs. One such technique that iteratively splits the original *d*-dimensional data space into $2^{d}$ sub-spaces is the quad-tree [139]. J. Berchtold et al. presented a pyramid-technique [133] to allow effective range queries. It is based on a unique Partitioning Approach and is optimised for high-dimensional datasets. The well known examples of space-based approaches are: iDistance, the R-tree family, Kd-tree, STR [140] and Quad-tree [139].

### 3.3. Dimensionality Reduction Strategy

Dimensionality reduction (DR) is a very popular strategy that has been widely used for high-dimensional dataset processing and it has also turned out to be a successful one. In real-life applications, the majority of important data are captured in the initial few dimensions only. Even though dimensionality could be reduced, the underlying data items might still have huge dimensionality because it is not necessarily possible to achieve this without losing important information.

A rapidly expanding volume of huge and high-dimensional data is used to create rich information in today’s data-based applications. Although the storage of this data is very common, properly indexing and retrieving it remains a practical issue. A kNN Search on these datasets is a common and expensive querying operation. Thus, to resolve this issue, researchers utilise the DR strategy prior to actually employing Indexing Techniques.

Based on Table 3 and Table 4, most existing approaches do not employ DR strategies. So, we indicated such techniques as “N/A”. The rest use the famous principal component analysis (PCA) technique or the iDistance (In addition to being a dimensionality reduction technique, iDistance is also an indexing method) technique.

**Principal component analysis.** PCA [78,80,81,141] is the most popular dimensionality-reduction technique used for transforming high-dimensional data space into lower-dimensional space [79,142]. It analyses the datasets to identify the directions that have the maximum variance and the one with the highest variance is considered the first principal component (or dimension). Then, the succeeding components capture the rest of the majority variance. Most of the information from the original space is captured in the initial few dimensions, where there is the highest variance.

### 3.4. Distance Computation Approach

Distance Metric plays a very important role in finding the *k* closest neighbour. So, for the given dataset in a *d*-dimensional space, we have to choose the best Distance Metric for the algorithm to work well. There are several Distance Metrics available, but we will just discuss those that have been used for the computation process in the kNN queries. The most widely used of all the Distance Metrics is the Euclidean distance function.

**Euclidean distance.** The distance between any two given data points can be calculated using many different approaches. One of the most well known and widely used approaches is the Euclidean distance (L2). A Euclidean distance (1) is a measurement of the actual straight-line distance in Euclidean space between any two given points. It is calculated as the square root of the sum of the squared differences between any given point (x) and another point (y).

(1) $$d\left(x,y\right)=\sqrt{\sum_{i=1}^{n}{\left(x_{i}-y_{i}\right)}^{2}}$$

**Bregman distance.** In a *d*-dimensional space, the Bregman distance between query point $q=(q_{1},q_{2},\ldots ,q_{d})$ and random data point $p=(p_{1},p_{2},\ldots ,p_{d})$ is defined as:

(2) $$D_{f}\left(p,q\right)=f\left(p\right)-f\left(q\right)-<\nabla f\left(q\right),p-q>$$

As demonstrated in an example [143,144], while matching pictures, the distance is not a metric measure. The sun and the ball both have a similar structure, yet they distinctly vary from one another. So, in some real-world situations, the Euclidean distance is not a good way to measure distance.

In recent years, in a range of systems such as image retrieval [145,146], image classification [147] and sound processing [148,149], the Bregman distances have been extensively utilised because they have the capability to explore the underlying correlations of data features.

During analysis, we observed that almost all the approaches use a Euclidean distance except the BP [114] technique. BP is a non-metric approach that uses Bregman divergences for distance calculations. The Bregman divergence is also known as the Bregman distance. This is the first non-metric work for the high-dimensional exact kNN search technique.

### 3.5. Computing Paradigm

In Section 4 and Section 5, we discussed the Computing Paradigm approach in detail to obtain an understanding of how all the existing techniques work. There, we classified the kNN Search (i.e., Section 4) and kNN Join (i.e., Section 5) techniques as I/O-based, Memory-based, Parallel and Distributed. Considering the length of the paper, it is very difficult to convey all the details of every work. So, you can also refer to their work to understand it in more detail.

## 4. kNN Search

In the majority of database applications, finding a similar object to the requested query point is an expensive job. The kNN query is ideally required in such circumstances. A great deal of research has been conducted to provide a solution to the exact kNN problem. However, most of the studies focused on approximation techniques. In addition, some researchers also consider the exact kNN strategies. For example, work such as HDR-tree, iDistance and others is proposed to address the issues of efficiently finding the kNN and reducing the cost of distance computation. In this paper, we mainly focus on the exact solutions. A comprehensive study of various high-dimensional kNN Search and kNN Join techniques is discussed. For ease of understanding, we have categorised the techniques in many ways. Here and in the next section, we summarise the most well known techniques and categorise them according to Computing Paradigms.

In this section, we concentrate on the various exact kNN Search approaches based on the different Computing Paradigms such as I/O-based, main-memory-based and parallel or distributed. We classified some techniques as memory-based techniques if the majority of the computation task is carried out by main-memory rather than disk-based I/O.

Several machine learning-based approaches, such as [150,151,152], are also proposed to address the problem of finding kNN in high-dimensional space. In [150], researchers presented a GPU-based kNN technique using CUDA-based radix sort [153]. The results show that this approach performs 30 times faster than the normal CPU-based approach. Here, they used a data segmentation approach for distance computation [154]. This approach involves using fixed-size tiles to create the segments and tile size is determined by the amount of shared memory per CUDA block, which is actually quite small. As a result, the approach involves a great deal of data access.

In [151], the authors provide a method to locate the specific kNN picture items that correspond to a particular query item. Basically, the suggested method first uses a self-organising map [155] algorithm to cluster the pictures and then it projects the identified clusters into points in a linear space depending on the distances between each cluster and a chosen reference point. These projected points are then arranged in an index structure known as an array-index. This method is very simple and compact in nature.

The new kNN technique named kMkNN (k-Means for k Nearest Neighbours) [152] was proposed to speed up the closest neighbour search process with the help of the triangle inequality and k means clustering. There are basically two steps involved in the kMkNN algorithm. In the building step, kMkNN preprocesses the training dataset using a conventional k means clustering algorithm. They divide the whole dataset into clusters using the k means clustering approach, and store the distance between every data item and its nearest cluster centre. Every cluster’s entire distances are then sorted in decreasing order. In the searching step, for a given query object, kMkNN employs the triangle inequality to decrease the distance computations during the searching stage by locating the closest data items, beginning with the cluster that is closest to the query object. We do not include learning-based approaches in a later discussion because they are outside the scope of our survey.

### 4.1. I/O Based

Earlier, algorithms were created to run in the main memory. Because of technological advancements and the large volume of data, it was difficult to fit the entire dataset within the main memory. This demands the development of more efficient I/O-based approaches.

**iDistance.** The researchers introduced a new index structure termed iDistance [110,111] to facilitate the exact kNN Search for HD data. It transforms high-dimensional space into a one-dimensional value. This process consists of three phases. It starts by segmenting the entire data space into m parts. Then, it assigns a reference point to every partition and finally transforms each partition into one-dimensional space based on other data points’ similarity to the reference point. Assume there are *m* partitions named $P_{1},P_{2},\ldots ,P_{m}$. The reference points for these partitions are named $C_{1},C_{2},\ldots ,C_{m}$ and are chosen based on the Data Partitioning or Space Partitioning strategy. Finally, all points $p_{i}=\left(v_{1},v_{2},\ldots ,v_{n}\right)$ are transformed into a one-dimensional data space. Here, they used two data structures: the B${}^{+}$-tree and the array. To enable quick retrieval, the transformed 1D points are indexed using a B${}^{+}$-tree. Additionally, an array is used to store the *m* reference points together with their closest and furthest radii. The secondary structure is first scanned in order to find the reference points whose data space intersects with the query region. The search starts with a smaller radius and it is then increased gradually. At first, the closest leaf node of query point *q* is searched and its sibling leaf nodes are also checked if required. The searching process stops when it finds the kNN for the query node and further increasing the query sphere has no effect on the closest list. Therefore, along with the right partition algorithm, iDistance can be considered one of the most effective kNN Search techniques. It cannot be neglected that the effectiveness of pruning algorithms decreases with increasing dimensionality and *k* value. However, this effect is less prominent for iDistance.

**Diagonal Ordering.** The researchers in this study developed a diagonal ordering strategy [112]. This strategy primarily relies on data grouping and sorting. High-dimensional data can be converted into 1D data by slicing the clusters diagonally. For indexing, they employed a B${}^{+}$-tree structure. The diagonal ordering is similar to the iDistance and Pyramid Technique. Here, the high-dimensional data space is divided into clusters and the vectors inside each cluster are arranged using the diagonal sorting order. In general, a cluster’s feature vectors are sorted first by partitions and then in the diagonal direction of every partition. The sorting technique provides a way to convert high-dimensional vectors into one-dimensional values. Then, to index these values, the B${}^{+}$-tree structure is utilised. This technique has the advantage of calculating a precise lower limit on the distance between two feature vectors using the diagonal order. It was seen that the kNN Search process improved when a lower limit was used as the pruning criterion to get rid of unnecessary feature vectors instead of doing expensive distance calculations. All the points on the line segment are assumed to be outside the search area if the minimum distance between a query point *q* and the line segment is greater than the search radius *r*. The diagonal order method also follows the iterative approach to search for the nearest neighbours. It starts with a small radius and gradually increases it until it finds the kNN. It stops the process when the distance between the query node and *k*-th NN is less than or equal to the search radius. To obtain an excellent order, they used PCA [141], emphasising the first few characteristics over the rest and used the clustering technique from iDistance (i.e., k means clustering). The performance of this technique was better than many existing techniques such as X-tree [136], iDistance [110] and VA file [71].

**VA${}^{+}$-file.** In ref. [109], researchers provide search methods that work particularly well with huge, high-dimensional datasets. Basically, they introduced an approach that is based on the scalar quantisation of data, which is known as the VA${}^{+}$-file. It is very helpful for finding kNN in non-uniform HD datasets. To further improve the search performance, they use the approximation technique. Here, they provide a general kNN approximate framework, talk about several methods for processing similarity queries and introduce a metric for measuring these methods. Lastly, a novel method based on clustering has been developed that combines the advantages of multiple methods for progressive similarity searches.

**OTI and EOTI.** In this study [113], using an optimum triangle-inequality (OTI) technique, researchers offer a novel fast kNN Searching technique. When searching kNN for every query point, the proposed OTI approach avoids the greater redundant distance calculations compared to the main TI technique. They also give an effective optimum triangle-inequality (EOTI) technique that is based on OTI and takes into account OTI’s large space and space complexity.

The fundamental concept of KMC-TI-FS (TI) [156] is to split the whole search process into two phases. In its first phase, every data item is grouped using the k means algorithm, which is known as the offline clustering phase. The TI approach is used in the online search process of the second phase to determine whether a particular data item is a probable k nearest neighbour of the provided query or not. Last but not least, the kNN Search can be sped up with very little distance calculation because the distance between all the items $p_{i}$ and the cluster centre $c_{i}$ was calculated and saved in advance in the offline clustering step, so no additional distance calculations were required.

The TI approach was able to eliminate the items in the central region of adjacent clusters but was unable to remove items from the marginal region. In order to use triangle inequality more effectively and solve the TI problem, they came up with a new quick search method called OTI, which stands for “optimized triangle inequality” [113]. The goal of a suggested OTI strategy was to choose an ideal cluster centre $c_{opt}$ from all of the cluster centres $c_{j}$ that help to make up the appropriate triangle. In TI, for any point $p_{i}$ it chooses its own clustering centre $c_{self}$ to form the triangle but in the case of OTI it chooses another optimal clustering centre $c_{opt}$ and typically $c_{opt}\neq c_{j}$ is true. The space complexity of this approach for distance storage is O(N×C), where *N* is the number of items and *C* is the number of clusters in the dataset.

The OTI approach resulted in very high space complexity and its search time performance was adversely affected by additional calculation time. So, it was essential to find a balance between search efficiency and space and time complexity. As a result, they propose an efficient optimal triangle-inequality (EOTI) [113] technique for locating an efficient optimum cluster centre $c_{eopt}$ with significantly reduced space and time complexity. In this approach, they simply record two distances namely $d(p_{i},c_{j})$ and $d(p_{i},c_{max})$ for each given item of $p_{i}$. Where, $c_{j}$ is the cluster centre closest to $p_{i}$ of all the given cluster centres and $c_{max}$ is the cluster centre that is farthest away from $p_{i}$. EOTI’s distance storage has a space complexity of O(N+N).

**BP.** Bregman distances, often referred to as Bregman divergences, are frequently used in voice recognition, machine learning (ML) and kNN Searches. Multimedia systems often turn original data such as audio, video and pictures into hundreds of dimensions. However, previous research on Bregman distance-based kNN techniques [135,144] was designed to deal with data of moderate dimensions (typically less than 100). Therefore, these index techniques were unable to perform well in high-dimensional space due to significant cluster overlap and costly computation operations. High-dimensional kNN Search with Bregman distances is a critical concern that is addressed in this study [114]. Here, they present a new partition-filter-refinement structure. This technique comprises precomputation and search processing. In precomputation, initially, they split an entire HD space into a number of small dimensional subspaces. Then, range queries are run over each subspace to obtain items. The kNN findings are then assessed via filtering of the items. However, in order to implement such a framework, they made the following contributions: On the basis of the Cauchy inequality, they compute the upper limits between the query point and any random data item within every subspace and the appropriate upper limits are chosen as the search limits from these subspaces. Additionally, a method known as Pearson Correlation Coefficient-based Partition (PCCP) is provided to minimise the item set by splitting correlated dimensions into several subspaces. To speed up the search procedure, they lastly used Bregman Ball trees (BB-trees) [135] in the subdivided low-dimensional subspaces and created an effective integrated, disk-resident BB-forest index structure. Then, create tuples from the data items to calculate the search limit. While conducting a search, they convert the query item into a triple [114] and then calculate the limit for the range query. Later on, they run a range query on the items. The kNN results from these items are then examined. Additionally, by balancing efficiency and accuracy, they transform the precise solution into an approximate BrePartition (ABP). The BrePartition is the first non-metric method that uses the Bregman distance and performs better in HD space when searching for the kNN.

### 4.2. Main Memory

The various researchers worked on disk-based approaches, considering that huge datasets cannot fit into the main memory. Due to the innovation and upgrade in the technologies, the remarkable fall in the RAM (Random Access Memory) prices and large storage sizes can be observed. This encouraged research interest in main memory-based approaches.

**Δ-tree.** To enhance the high-dimensional queries in main memory, researchers proposed a new index structure called the Δ-tree [74]. It has a multilayer structure where dimensionality increases monotonically from root to leaf level, i.e., every level provides the data space at a varied dimensionality with the help of PCA. Every level of the multilayer tree helps to minimise the search area since the lower dimensions speed up distance calculations and make better use of the short cache line size. The Δ-tree helps to prune the search region very efficiently. As per the PCA property, the distance between any two points in the PCA-transformed lower dimension will always be lower as compared to the higher dimension. As a result, if the distance between any data item and the query point in low dimension is greater than the original distance of the existing *k*-th nearest neighbour, it can be discarded. In order for a tree to work well, it must have the right number of levels and dimensions at each level. However, the Δ-tree suffers from a few limitations: 1. Its effectiveness depends on how well a dataset is globally correlated (i.e., it works well for correlated data). 2. It needs to process the whole dataset to find the PCA eigenmatrix. 3. The whole tree needs to be rebuilt on a regular basis to improve its overall performance.

**array-index.** Researchers proposed an array-index [117] plug-and-search technique in order to enhance the kNN Search performance of the Data Partitioning Approaches on real datasets (i.e., very skewed and correlated) while maintaining their features. This method reads the data partitions generated by the high-dimensional Data Partitioning Approach and linearises the partitions with the help of ordering. It is sorted by the distance between the chosen reference point and the representative vector of each partition. The resultant computed distance helps them to map the partitions to 1D array-index space. As a result, related partitions are brought close to one another, enabling them to develop a faster method to discover the kNN answer points. Thus, for any given query *q*, the proposed algorithm has to look for a very small region. The results show that plugging the array-index into a Data Partitioning Approach significantly improves the kNN Search time.

**$\Delta^{+}$-tree.** To address the limitations of the Δ-tree algorithm, they proposed an improved version, known as the $\Delta^{+}$-tree [74,118]. The core concept of $\Delta^{+}$-tree was first introduced in their earlier work [74]. They have provided a detailed index approach as well as dynamic update techniques in this work. To overcome the first constraint of the Δ-tree, they globally split the data space into many clusters and used PCA for every cluster separately. To deal with the second limitation, they divided the cluster into smaller segments based on its distance from the centre, which helps to reduce the number of areas that must be evaluated. Finally, they construct a Δ-tree for every segment. It helps to minimise the computational cost and cache misses.

**ACDB.** For high dimensional indexing, Hong et al. propose an enhanced kNN Search method based on adaptive cluster distance bounds [119] by lowering the CPU cost using the triangle inequality. They stated the two key algorithms: the kNN Search algorithm and the generation of Voronoi clusters. For cluster indexing, initially, the entire dataset was divided into many Voronoi clusters, which were separated by the hyperplanes. The distances between each Voronoi cluster and all of its hyperplanes were then determined. These distances are then put into a file. The Euclidean distance measure is used to index the items in each cluster. Essentially, each kNN query $q_{i}$ determines the lower distance limits for each cluster dynamically and then the clusters are sorted in ascending order. Afterwards, it searches for the kNN from a cluster when it is loaded into memory. Then, with the help of kNN distance, it checks whether the maximum distance of kNN is less than the next cluster’s lower distance limit. If it is, then it finishes the search process. If not, then it loads and checks the following ordered clusters using the same strategy. They adopt the triangle inequality to further speed up finding the closest items in a cluster and lower the CPU cost.

**iDistance-PS.** In [120], researchers undertook the first extensive study of several partitioning techniques for the iDistance method. They demonstrate how the performance of iDistance is significantly impacted by partitioning techniques and also discuss the state-of-the-art for utilising the indexing approach in current applications or comparative assessments. Since its first release, iDistance [110,111] has become one of the most effective and cutting-edge high-dimensional indexing approaches. Recently, it has been employed in a variety of challenging applications, including image retrieval [157], video indexing [158], etc. [159,160,161]. In this work, they also provide an open-source version of the original iDistance technique (http://code.google.com/p/idistance/) (Accessed 22 November 2022). The Partitioning Approach can be considered a significant computational parameter for iDistance. Here, the authors mentioned the three fundamental stages for processing a query *q* of radius *r* are as follows: Initially, it identifies the set of divisions to search and then it needs to figure out the search range for every division in the set. Finally, the data points are retrieved and then filtered by actual distance.

**PL-Tree.** In ref. [115], the authors introduced a novel Indexing Technique to facilitate effective point queries, range queries and kNN queries. The PL-tree technique recursively divides the original data space into hypercubes until it has a specific number of data points in it. They are labelled using the Cantor pair function where items within the same hypercube end up with the same label. Because of the Cantor function’s computational effectiveness and bijective property, high-dimensional vectors can be easily mapped to scalar labels. If the number of data objects in a subspace exceeds its limit, the partitioning and labelling procedure divides the subspace.

**iDStar.** In this study [162], many important and manageable factors are looked at in order to improve the efficiency of kNN Search queries using the iDistance and iDStar algorithms. They also show the challenges of indexing in high-dimensional and tightly-clustered dataspaces. Through experiments, they discovered that the iDStar method of local division always works better than the iDistance method in any clustered space with fewer than 256D. Ref. [162] is based on earlier evaluations of iDistance partitioning techniques and iDStar extensions [120,163,164].

In earlier research, it was demonstrated that the iDistance effectiveness remains stable in high-dimensional and closely clustered spaces by retrieving a complete partition to fulfil the requirements of a specific query [163]. In iDStar [115], researchers additionally divide the dense regions of the dataspace by dividing partitions into different parts that correspond to separate parts of the B${}^{+}$-tree, which could be arbitrarily pruned during the retrieval process. To accomplish this, they modified the indexing and retrieval methods in various ways, which indirectly affects the way we use the B${}^{+}$-tree. Initially, the mapping function was revised to construct a continuous region division inside the already separated divisions. Secondly, once they have determined the region they should look for, they have to identify parts inside every region that needed to be searched, along with their updated search ranges within the B${}^{+}$-tree. They also store a sectional $distmax_{i}^{j}$ (for partition $P_{i}$ and section *j*) during index construction. Here, $distmax_{i}^{j}$ is the distance to its farthest point. In experimentation results, it can be seen that all data fit in the memory, which avoids the usual I/O bottleneck problem.

**HC-O.** In order to accelerate the item filtering process during the kNN Search, they introduced a caching compact approximation [116] of data point renderings in the main memory. However, it exhibits two complex problems: 1. Which data point encoding strategy is the most efficient for supporting kNN Search? 2. How many bits should be used to encode a data point? For the first problem (1), they develop and resolve a new histogram optimisation problem that determines the best encoding method. They also proposed a cost model for (2) in order to automatically modify the optimal number of bits for encoding items. The proposed strategy works for both exact tree-based indexes and approximate LSH techniques, so it can be used by anyone. Here, they also offer a way of accelerating kNN Search on tree-based indexes. They basically started by running queries and collecting information about how often each leaf node is accessed. Then, leaf nodes are added to the cache in decreasing order of the access frequency. Lastly, using an efficient histogram construction method [116] they create the histogram *H* and calculate the approximate representations of data points (in leaf nodes). This proposed cache can be used for any tree-based kNN Search solution (for example, [111,165]), with a few small alterations. The proposed caching technique HC-O turns out to be way better than the exact caching (such as iDistance, VP-tree [165] and VA-file [166]).

### 4.3. Parallel and Distributed

In the past few years, faster computing has led to a rise in the number of diverse datasets in all fields. For a general sequential method, handling high-dimensional datasets is a very costly and time-consuming operation. As a result, few works employ parallel and distributed computing techniques to speed up processing. In order to manage a high-dimensional dataset in the main memory associated with the processors, the use of multiple processors makes it possible to utilise additional memory. Thus, parallel data processing is highly sought after in a variety of applications. The recent availability of GPUs for general purposes opens a door for parallel processing. Using the NVDI CUDA API greatly improves performance. It provides a powerful platform for parallel processing functionality.

**BF-CUDA** Computing a kNN across huge collections of *d*-dimensional vectors is a computationally expensive operation. Pre-structuring the data, such as by utilising binary trees, helps lessen this computational cost. This work [77] focuses on the CUDA implementation of the kNN Search using brute force (BF). BF is a two-step process, i.e., distance computation and sorting. One of the most common and simplest ways to look for the kNN is the BF approach. In the BF algorithm, the process of searching the kNN for a given query *q* is as follows: 1. Determine the distance between all data points $s_{i}$ for query point $q_{i}$. 2. Using the calculated distance, sort the neighbours in ascending order. 3. Determine the *k* closest neighbour points for a given query point. 4. Repeat steps 1–3 for each query point. For experimental purposes, they used the variant of an insertion sort, which is faster and better than the comb sort for small values of parameter *k*. The BF approach is extremely parallelisable by default, which makes it well suited for a GPU implementation. Global memory and texture memory are the two types of memory utilised. Despite the global memory’s large bandwidth, the performance drops if memory accesses have not coalesced. The experimental results demonstrate that using the NVIDIA CUDA API speeds up the kNN Search up to 400 times the speed compared to using a CPU-based BF approach.

**CUBLAS.** The implementation of the kNN Search was built using CUDA, and CUDA Basic Linear Algebra Subprograms (CUDA implementation of BLAS, i.e, CUBLAS) [75] consist of the following kernels: Coalesced read/write calculations for the vectors $N_{R}$ and $N_{S}$ were performed using CUDA in steps 1. and 2., respectively. In step 3., CUBLAS is used to calculate the *m* × *n*-matrix $A=2R^{T}S$. 4. Using CUDA, add the $i^{th}$ element of $N_{R}$ to each element of the $i^{th}$ row of the matrix *A*; the resulting matrix is denoted as *B*. 5. Use the insertion sort variant [77] to sort each column of *B* in parallel; *C* represents the resulting matrix. 6. Using CUDA (coalesced read/write), add the $j^{th}$ value of $N_{S}$ to the first *k* elements of the $j^{th}$ column of the matrix *C*; the final matrix is denoted by the letter *D*. 7. To obtain the *k* lowest distances (coalesced read/write), square the first *k* components of *D*; *E* is indicated as the resultant matrix here. 8. Remove the top-most k×n-submatrix from *E*; the generated matrix is the required distance matrix for each query’s *k* nearest neighbours. CUBLAS does the main work of computing, which is the calculation of *A* in kernel 3. On synthetic data, the CUDA and CUBLAS implementations were up to 64 times and 189 times faster, respectively, than the highly optimised ANN C++ library. Additionally, they provided the open source code (https://github.com/vincentfpgarcia/kNN-CUDA) (Accessed 22 November 2022).

**TBiS.** Numerous initiatives have leveraged GPUs (particularly those made by NVIDIA) as multi-core parallel processing units with growing support for application interfaces [75,150]. The majority of GPU implementations adapt or customise certain sorting algorithms as needed. As such, Garcia et al. used the insertion sort as well as a parallel comb sort [75]. Because the process of reading, comparing, swapping and writing for sorting is data-independent, bitonic sorting is well suited for parallel systems [167]. In this work, Sismanis et al. [122] propose a novel study into parallel methods for determining the kNN of each individual query in a high-dimensional space on a multi-core processor that supports synchronous processes, such as a GPU. They focused on the BF kNN sorting process and presented a group of truncated sort algorithms for parallel kNN Search by utilising the close link between the two fundamental operations of select and sort. In particular, the truncated bitonic sort (TBiS) offers simple data and programme structures, effective data locality and synchronous concurrency. They outline several techniques and demonstrate that their truncated bitonic sort performs extremely well on the GPU. The overhead of TBiS decreases with each successive iteration. The time complexity of the parallel scan was O(logn). Here, they first identified the *k*-th element as a threshold. Then, all elements below the threshold are examined and further searches are conducted to find elements that equal the threshold. Whenever it becomes certain that an item cannot be a part of the minimal *k*, it is removed from the sort. Among the methods mentioned here, two methods, Bubble Sort and Bitonic Sort, have data-independent synchronous processes.

**QDBI.** In the context of peer-to-peer (P2P) systems that manage and process a growing amount of high-dimensional data such as text, photos and videos, understanding how to search through this data has increasingly become a key research question. The processing of difficult queries over high-dimensional data items, such as the kNN query, has been the subject of several studies [168,169,170]. For effective data management and query optimisation in a P2P system, in this study, researchers present a distributed multi-dimensional data index (QDBI) [125] which is based on quad-trees. Here, every peer uses an MX-CIF quad-tree to generate an index item for their high-dimensional data. Every index item then obtains a code in accordance with the MX-CIF quad-tree. The entire index is then organised into 1D rings based on their codes. Super-peers dynamically join the rings in accordance with the requirements. This creates a structured super-peer network that is based on semantics and an effective kNN query processing technique is designed for high-dimensional data items based on the QDBI index structure. Research demonstrates that the kNN query method and the index structure both offer good search performance and scalability. Whenever a super-peer gets a kNN query, it first routes it to the super-peer that stores its associated control point (quad-tree block), after which the super-peers evaluate the query simultaneously. It might obtain the complete solutions from one super-peer to multiple other super-peers along the ring in the counterclockwise direction because the index items are stored in the ring clockwise according to the code value of their control points. The concept is that the search space is expanded when the query is extended from a narrow range. Finding the *k* closest data points to the query point in each search space allows it to update the existing data in all search spaces with the more recent data. When the closest data quantity exceeds *k* and does not get any closer over time, the whole process comes to an end.

**CU-kNN.** In ref. [121], researchers emphasised CUDA architecture-based GPU parallelisation of data mining applications. They originally offered three CUDA-based parallel algorithms to take advantage of the novel parallel platforms for data mining: 1. A scalable threads scheduling strategy for irregular patterns, which is used to address the problem of irregular pattern computing; 2. a parallel distributed top-k strategy, parallel selection of top-k values; and 3. parallel high dimension reduction using the dimensionality reduction technique. Then, utilising the aforementioned methods and the CUDA platform, they built the three common data mining algorithms: CU-Apriori, CU-kNN and CU-K-means. These suggested methods enable CUDA versions of these algorithms to operate effectively. However, because our research focused on kNN approaches, we will discuss the CU-kNN approach only.

In CU-kNN [121], it has been seen that the majority of kNN computation is found in two cores: choosing the kNN and calculating distance. Here, they demonstrate the CUDA implementation. There is no data transmission between host memory and device memory throughout the classification process in this approach since the GPU handles all the calculations. Because pair-wise distance computation is independent, it is possible to parallelise the process entirely. With this characteristic, kNN is well suited for a GPU-parallel implementation. The objective of this kernel is to reduce the number of global memory accesses while maximising the concurrency of the distance computation executed by several threads. They split every object into many parts based on a dimension with a specified size to handle the multi-dimension property of objects in this application and then they iteratively calculate the distance between each of the partitions. The cooperative efforts of the threads load one feature segment of the query objects and the associated feature segment of the reference objects into the shared memory at a time via a coalesced read and then perform a local calculation to generate a local summation. These local sums are added together to determine the ultimate distance value. The main goal of choosing the *k* nearest neighbour of a query item is to find the *k* shortest distance, which is a standard top-*k* issue. Therefore, they put it into action using the parallel distributed top-*k* approach. Since choosing the kNN for different query objects is independent, they do it in parallel by giving some threads the job of choosing the *k* shortest distances for a single query object. The tests revealed that CU-kNN outperforms an effective Fast-kNN [77] by up to 8.31 times on the KDD-CUP 2004 quantum physics real-world dataset.

**kNN-PA.** In this study [123], the authors propose techniques and a library for performing closest neighbour searches on any high-dimensional dataset across thousands of cores using the message passing interface (MPI) [171] and OpenMP [172]. The proposed library provides exact and approximate solutions. This study is mostly about a method for solving approximate nearest neighbours (ANNs) which uses tree indexing, but here we will concentrate on kNN approaches only. They have introduced two distributed brute-force kNN techniques, which are two-dimensional partitioning and cyclic partitioning techniques. In the first approach, initially they partition the reference and query points into parts and move them to the nodes. Due to the fact that the reference points and the query points are replicated multiple times, this approach is more memory intensive as compared to cyclic partitioning, but when estimated time cost is an essential performance parameter, this approach is much more helpful. The second technique, which requires more communication but is memory-optimal, employs cyclic iteration. This approach divides *R* and *S* into roughly equal-sized segments that are distributed across the processes. Based on which set is bigger, either the reference points or the query points can be cycled.

Researchers also introduced the parallel tree-building technique called RKDT (randomised k dimensional tree) [123] for indexing structures in any number of dimensions. This method supports different kinds of trees, such as ball trees, KD-trees, etc. The majority of tree algorithms for high-dimensional datasets use a top-down approach. The tree is used to divide and filter spatial searches. They investigated two point-splitting methods: cluster-based (also known as $RKDT_{C}$) and hyperplane-based ($RKDT_{H}$). For the cluster-based method, they utilise k means clustering and a standard KD-tree is used for hyperplane splitting. In a hyperplane-based technique, they compute a projection direction and project all points onto it. They try it with the three potential projection choices. The first is random selection. Another is selecting the coordinate that has the greatest variation among all the points. At last, they chose the direction in which the points are furthest apart. According to the researchers’ experimental results, they find that the partitioning based on the hyperplane is better than the cluster-based approach. Using the hyperplane strategy, they can equally divide the data points, yielding nearly the same number of points per group, which has significant effects on the parallel implementation and load balancing of the method. On the other hand, clustering-based partitioning is more costly as it requires solving k-means problems again and again. They also find that improved filtering during neighbour searches is achieved by the hyperplane splitting technique. The two most common tree traversal techniques are greedy traversal and bounding ball traversal. In a greedy traversal, a query point traverses the current node’s one of the child nodes. On the other hand, in bounding ball traversal, the query node visits each leaf node that intersects the bounding ball. To overcome the kNN challenges, they used the two-stage tree traversal approach. In the first phase, every query point uses a greedy traversal to reach a leaf node and then find its kNN. Then, in the second phase, all nearest neighbours within the bounding ball are found using a bounding ball search with a radius equal to the kNN (i.e., *k*-th item distance). Finally, among all detected neighbours, the closest *k* items are returned. In a high-dimensional dataset, the bounding balls and leaf nodes overlap significantly.

**HkNN.** In ref. [124], the authors implemented a hybrid approach for kNN Search over the high-dimensional dataset. D. Muhr and M. Affenzeller noticed that the indexing structures that are usually used for kNN Searches in high-dimensional spaces do not work well and therefore they find that there is a need for efficient brute-force search. Using a highly parallel method for brute-force search, the proposed method divides the computing work between the CPU and GPU in an effective way. The main objective behind their technique was to examine how the massively parallel architecture GPGPU paradigm [173] and CPU-based shared-memory parallelism work together to overcome the problem of dimensionality in kNN Search. Here, the CPU carries out k selection asynchronously while the GPU performs distance calculation. Through the use of batching techniques, the distance calculation on the GPU and the k selection on the CPU are performed concurrently. They show that the effectiveness of their technique increases linearly as the number of dimensions of the dataset increases, making it more efficient as compared to other methods for large datasets. This approach was developed in Julia and is open-source (https://github.com/davnn/ParallelNeighbors.jl) (Accessed 22 November 2022).

## 5. kNN Join Approaches

Spatial joins are used in many existing high-dimensional join processing techniques. It was created for 2D objects. In the literature, several spatial join approaches have been presented [174,175,176,177]. It is also worth noting that similarity join methods [178,179] have been developed to find all paired objects that are closer than a user-specified distance. However, these techniques to process similarity join cannot be effectively applied to kNN Joins since it is hard to predict the search radius in kNN Joins [180,181].

If we look at the existing studies, there were mainly two types of similarity join, i.e., distance range join, also called range join or distance join and k Distance Join, which is also known as closest point query. In [53,54], Böhm and Krebs introduced the third type of similarity join, which is known as the kNN Join. Incremental distance join is also sometimes considered a type of similarity join.

We further classified the different kNN Join approaches into I/O-based, main-memory-based, parallel and distributed techniques.

### 5.1. IO-Based

In the literature, many studies have been carried out on index structures that can handle kNN Join. For instance, Böhm et al. [54] presented a kNN Join problem, which finds the kNN for a set of queries in a single run operation. The work on kNN Join techniques such as MuX (Multi-page Indexing) [54], Gorder (G-ordering kNN) [126] and iJoin [128] involved the nested loop searching strategy for high-dimensional datasets.

**MuX.** Böhm and Krebs [53,54] presented the first work on kNN Join. To compute the kNN Join, the researchers proposed a novel approach called multi-page indexing, which uses the index nested loop join approach and follows the R-tree [61] structure. So, to minimise the I/O time, large-size pages (hosting pages) were used. It also uses buckets, which are smaller minimum bounding rectangles, as a secondary structure to split the data more precisely and with less CPU cost. An index is provided for every set of objects (i.e., *R* and *S*) and then MuX iterates the index pages on *R*. For pages of *R* maintained in the main memory, pages of *S* are fetched using the index and search for the kNN. When all pages have been visited or filtered out, the process reaches its end. A combination of page-loading and bucket-selection strategies enhanced MuX performance. However, this approach has certain limitations, i.e., performance degrades with an increase in the dimensionality and high memory overhead that restricts the scalability of MuX kNN Join.

**Gorder.** The Gorder (G-ordering) [126] is a block-nested loop join approach. It utilises sorting, join scheduling and distance computation to reduce both I/O and CPU costs. It introduces two different phases, i.e., PCA [78] and grid order sorting. PCA identifies the direction in which the data have more variance. The Grid Order sorting splits the entire dataspace into rectangular cells. After partitioning, it is ordered according to the distance of blocks and applies the scheduled block nested loop join on G-ordered data. Gorder splits the G-ordered input dataset into blocks which consist of many physical pages. Two characteristics define the join stage of Gorder. Firstly, it uses a two-tier partitioning method to optimise I/O and CPU times independently. In a subsequent step, it schedules the data joining to enhance kNN processing. In the first-tier partition strategy, for every *R* block, it searched for the nearest neighbour from the loaded *S* block. *R* blocks are loaded sequentially and iteratively in main memory and for each loaded *R* block an *S* block is also loaded based on the scheduled sequence of similarity. The high CPU cost is incurred because of the large block size. Hence, for more efficient processing, a second-tier partition strategy is used. In the main memory, it splits the blocks into sub-blocks. As per the experimental result, one sub-block contains 20–25 points for efficient processing. Because of the high-dimensional data, reduction of distance calculation is critical for CPU time optimisation. Therefore, they devised an algorithm to reduce the distance computation. It first calculates the minimum distance between two blocks of G-ordered data, $B_{r}$ and $B_{s}$, and checks whether it is greater than the pruning distance. If it is, then it is to be pruned. If it is smaller than the pruning distance, then it is considered the *k* nearest neighbour.

**iJoin, iJoinAC and iJoinDR.** The proposed iJoin [128] method follows the iDistance index structure and the properties of the iDistance partition strategy help it to significantly improve the performance. Here, they have come up with three different approaches: the basic approach (called iJoin) and its two improved versions (i.e., iJoinAC and iJoinDR). In the first extended version, approximation bounding cubes were used to minimise unnecessary kNN computation and disc usage (iJoinAC). In later improvements, the reduced dimensions of the data space were used to minimise I/O and CPU costs (iJoinDR). In iJoin, the two datasets *R* and *S* are divided into clusters with the same reference points. To index the datasets, two B${}^{+}$-tree based iDistance indexes were constructed. The join process was initiated by considering the partitions of *R* and *S* which are within the search radius. The remaining partitions are pruned. If desired join pairs are found, then it stops the process. Otherwise, they widen the search area and look for more *S* nodes. The main objective of iJoinAC was to lower the number of distance calculations. If we exclude the leaf level navigation section, the iJoinAC method and the iJoin algorithm are nearly identical. Rather than processing the original feature vectors, it works on approximation cubes. It was observed that the processing approximations were faster than the processing of real feature vectors. They also present another improvement that utilises dimensionality reduction i.e., iJoinDR. This improvement is basically based on two methods. Using the dimensionality reduction technique PCA, the majority of the information is captured by the initial dimension. Secondly, the sorting method is considered a very efficient option for many issues. This approach is also similar to iJoin, but here the initial vector which captures the majority of the information has been used for the approximation and filtering process.

However, [54,126,128] performed the operation on a static dataset, so when updating it they have to perform the kNN computation on all the users, which is a very costly process.

**IIB and IIIB algorithm.** In ref. [127], researchers proposed the following three kNN Join algorithms for high-dimension data. These algorithms perform well for the sparse dataset. The BF algorithm compares the similarity score of each query point *r* in block $B_{r}$ to the similarity score of each object point *s* in block $B_{s}$. If the similarity score turns out to be higher than the pruning score of *r*, then the object point was considered as the nearest neighbour to the query point. Furthermore, it updates the pruning score after every update operation. The inverted index-based algorithm (IIB) is used to avoid unnecessary traversal of every item in the object dataset. This algorithm helps to overcome the issues BF was facing. For every feature of query point *r*, rather than visiting all, it prunes the unnecessary features of *s* during the calculation of the dot product of *r* and *s*. Here, they utilise the inverted list $\left\{I_{1},I_{2},\ldots ,I_{D}\right\}$ which is a set of lists (for every dimension). It computes the kNN for each *r* query point in $B_{r}$. An improved inverted index-based algorithm (IIIB) enhances the features of IIB. The authors came up with a new threshold-based pruning algorithm called IIIB. It uses the previously calculated results as a threshold for the next loops. These approaches resolve the kNN Join problem for sparse vectors.

**kNNJoin${}^{+}$.** Yu, Cui, et al. introduced the kNNJoin${}^{+}$ technique for processing kNN Join queries on high-dimensional data. The RkNN query is an extremely expensive process as compared to kNN. As a result, it can be regarded as optional, meaning that it can only be utilised when necessary. Tables are dynamically updated for all operations. Four different types of data structures were used in this study: the RkNN Join table, kNN Join table, iDistance and sphere-tree. The sphere-tree is used to look for RkNN, i.e., points with *p* as their kNN, whereas the iDistance indexing is used to find the kNN for a newly inserted point *p*. The Pyramid technique is used by the iDistance method to transform a high-dimensional space into a single-dimensional value. It divides the data space into *m* parts, assigns a reference point to each partition and then converts each partition into a one-dimensional space based on the similarity of other data points to the reference point. These values are then mapped to the B${}^{+}$-tree so that they can be accessed and updated more quickly. They develop a shared query optimisation strategy in order to improve performance.

### 5.2. Memory-Based

**HDR-Tree.** Here, Yang, Chong et. al. [83] provided two different data structures, namely HDR-tree (exact) and HDR*-tree (approximate solution). The HDR-tree utilises the PCA [79] and clustering approach for dimensionality reduction. On the other hand, HDR*-tree employs the Random Projection [182] method. It uses a random matrix to transform the datasets from *d* to *r* dimensions. The PCA approach is basically used to reduce the cost of computation in the tree structure. Consider the $X_{N.d}$ dataset, which has been transformed from *d* to *r* dimensions. During the process of reducing the number of dimensions, the direction with the most variation was chosen as the first principal component and then the second. The first dimension of the tree structure consists of values with high variance. Using different dimensions gives better pruning power, which helps to reduce the computation overhead. With the help of eigenvalues (derived from the covariance matrix of the input dataset), they use different dimensionality at different levels. The high-dimensional dataset was partitioned into clusters using the k means clustering approach. The root level was set with $d_{1}$ dimensionality and at the next level, dimensionality $d_{2}$ was set. Every cluster from the root node was sub-clustered. At each level, the dimensionality increases monotonically. The leaf node was at full dimensionality $d_{l}$, at level *l*. The HDR-tree search algorithm looks for the affected users in a leaf node. It directly computes the distance between the user and the item in the leaf node (LN). On the other hand, non-leaf nodes look for the pruning condition and continue the search process if it is met. If the distance between the transformed item and the cluster is greater than the maxdknn value (i.e., $dist_{pca}\left(i,C_{j}\right)\geq maxdknn$), the cluster is pruned because users within the cluster are not affected by the item’s update operation. Thus, its child nodes need not be visited further. Here, maxdknn is the maximum distance between the users and their kNN. The HDR-tree [83] method searches for the affected users caused by any update operation and updates the kNN result. It addresses the issue of continuous kNN Join processing over high-dimensional real-time datasets. This turns out to be a very useful approach for reducing in-memory search costs.

**CTD-kNNJ.** Data become less relevant to some users as time passes. So, it is important to suggest the most recent data for the query object that was asked for. In order to deal with these problems, the researchers provided a time-dependent kNN Join solution [183] that could be applied to any distance function. In this study, the exact solution was computed by the kNN Join dynamically. Time is the critical parameter that has to be considered. When the number of dimensions goes up, say to 4096D, the normal distance function will be very expensive to use. So, they used an approximation strategy that helps to reduce the distance computation with the help of the Hamming distance technique. These techniques can be applied to all kinds of time-dependent distance functions. They stated two approaches for the time decay process, i.e., intersectable and non-intersectable. They mentioned the algorithm for time-dependent exact kNN Join but did not provide any improvisation technique for exact solutions.

**EkNNJ.** Real-world applications often perform dynamic update operations upon every insertion or removal of an item. However, as per the analysis, it has been observed that fundamental operations like batch updates and efficient deletion operations are not supported by the current existing kNN Join algorithms. Therefore, the authors of [129] present a novel approach for kNN Join over high-dimensional datasets where they focused on batch operations, lazy updates and optimised deletions. The results of an experiment show that the proposed approaches outperform existing techniques such as HDR-tree and naive RkNN. Whenever any update operation occurs, i.e., insertion or deletion of an item, they initially identify the affected users and mark them as “dirty” nodes in the HDR-tree. So, in lazy updates, the actual update is delayed until the kNN values of the affected users are needed. They also observe that the many newly inserted items affect the same users again and again, which causes redundant computation. To avoid this costly operation, rather than updating the user for every new item, they perform batch operations. They look for affected users but do not update them with each newly inserted or deleted item. Actually, to minimise computation costs, they process all updates at the node left, before updating the parameters at the internal level. Performing a deletion operation in kNN is a very expensive job. Normally, for any deletion operation, all the affected users need to be searched (i.e., RkNN process) and, accordingly, their kNN list is updated, which is a computationally expensive operation. To reduce the search cost and accelerate the process, they maintained a reversed kNN (RkNN) table for all items in the proposed method.

### 5.3. Parallel and Distributed

Considering the high-dimensional data, kNN Join is a very costly operation. To deal with it, various efficient approaches have been proposed but they were designed to run on a single machine or single-threaded environment. To tackle the issue, parallel and distributed approaches catch the researcher’s attention. It has been accepted widely not only in industry but also by academics. For this purpose, MapReduce [184] is the most widely accepted framework. It is famous for its simple but effective parallel and distributed computing model. The main purpose of the Distributed system is to overcome the limitations of main memory (i.e., ineffective in processing huge amounts of data).

Researchers have developed parallel MapReduce kNN Join algorithms, namely H-BNLJ [130], H-BRJ [130] and PGBJ [58]. The MapReduce [184] model is basically used to process a huge dataset in parallel in a very efficient way.

**H-BNLJ and H-BRJ.** In this work, researchers proposed a Block Nested Loop Join (BNLJ) [130]-based normal approach and further improvised the work using an R-Tree-based index (i.e., H-BRJ) [130]. It was observed that the basic approach is unable to scale well for high-dimensional and multidimensional data. Therefore, they came up with a new approximation algorithm that maps the multidimensional data to one dimension using space-filling curves (z-values).

They followed the simplest way to implement kNN-joins in MapReduce by using the block nested loop join technique. Basically, they divide the *R* and *S* datasets, respectively, into *n* equal-sized blocks in the mapping phase. This was accomplished quickly by linearly scanning *R* (or *S*) datasets and grouping each |R|/n (or |S|/n) record into a block. After the mapping phase, each potential pair of blocks (one from *R* and one from *S*) is divided into buckets. Then, one *r* reducer is run for each bucket the mappers created. Each reducer reads a bucket and runs a block nested loop kNN Join between the *R* and *S* blocks in that bucket or uses a nested loop to locate kNNs for every record in the local block of *R* from the local block of *S*. All reducers’ resultant output is written to DFS (Distributed File System) files. They just store the records’ ids, and the distance between each record r∈R to each of its kNNs from a local *S* block. The record output format is (rid,sid,d(r,s)). Each reducer obtains a pair of (rid,list(sid,d(r,s)) in order to sort the list (sid,d(r,s)) in ascending order of d(r,s). The top-k results for each rid are then released by the reducer. This technique is called H-BNLJ (Hadoop Block Nested Loop Join).

To improve the H-BNLJ strategy, researchers build an index for the local *S* block in a bucket in the reducer, so that it can facilitate the kNN Searching of the record *r* in the same bucket. They first use the R-tree to build a reducer-local spatial index over each block $S_{bj}$ and then they discover the local kNNs for each record from the local *R* block in the same bucket as $S_{bj}$. Furthermore, they respond to $knn(r,S_{bj})$ in each bucket of the reducer using the kNN feature of R-tree. Since bulk loading an R-tree for $S_{bj}$ and kNN Search in an R-tree are both highly efficient, the cost savings from not performing a local nested loop in each bucket more than balances this expense. The remaining phases are adapted from the H-BNLJ similarly. Therefore, they refer to this approach as H-BRJ (Hadoop Block R-tree Join).

**PGBJ.** PGBJ [58] is a partitioning and grouping technique that uses the Voronoi diagram to partition the entire data space into several cells and allocates the data based on the closest pivot in each cell. It does not always guarantee to provide the kNN result from within a single cell. Therefore, there is a need to look for more than one cell, which results in duplication and extra-distance computation. Here, the author has provided two different approaches for grouping the cells into large cells. (1) Geo grouping, which considers that the cells closest to each other are more likely to be duplicated and (2) Greedy grouping, which looks at the cells that seem to have the highest chance of being duplicated. It was observed that increasing the *k* value does not affect the communication overhead. Moreover, disk usage is also very low in PGBJ. However, this approach is inefficient for the high-dimensional dataset, which is its main drawback. Selecting pivots has a significant impact on the performance of PGBJ and it is also a time-consuming operation in terms of big datasets.

## 6. Applications

kNN queries are associated with a wide spectrum of applications. We list some examples as follows.

### 6.1. Sensor Networks

Applications in sensor networks include intrusion detection systems [6,7], fault detection [8], fault identification [9], fault classification [10], fall prediction [11], indoor localisation [12,13], etc. The intrusion detection system [6] can distinguish between unusual and common nodes by monitoring their anomalous actions. In [8], it addresses the gas sensor arrays fault detection issue, i.e., it can be employed in mine for monitoring and to provide an early warning.

### 6.2. Robotics

In the field of robotics, it is used for arm movement recognition [14], human emotion classification [15,16], scan matching [17], object recognition [18], fast point cloud registration [19], etc. The goal of arm movement recognition [14] research was to improve classification using kNN for various prosthetic arm motions. In [17], the authors performed scan matching with the help of the iterative closest point (ICP) algorithm. The purpose of scan matching is to match two misaligned scans from a mine tunnel using ICP. It recovers the relative position and orientation of two laser scans.

### 6.3. Mining Industry

All these applications of robotics and sensor networks are used in the mining industry. Along with it, in the mining industry, kNN queries are also used to predict blast-induced ground vibration in open-pit coal mines [20], safety risk assessment and risk prediction in underground coal mines [21], classification of human activities (such as lying, sitting, standing and walking) [22] and the Iterative Closest Point (ICP) approach for calculating the similarity of 3D log scans in the wood industry [185,186], etc.

### 6.4. Recommendation Systems

The kNN is a popular approach in recommendation systems. Based on what the user selects, we can suggest a similar item. This makes it more likely that the user will like the item. It is applicable for recommending products, recommending media to users and also for showing targeted relevant advertisements to customers. Many well known companies offer personalised recommendations to their customers, including Netflix, Amazon, YouTube, Spotify and many others.

### 6.5. Data Mining

The kNN technique is also widely used in data mining fields such as pattern recognition [26,27,28,29], regression [30,31,32], outlier detection [33,34,35,36] and others because of its simplicity, demonstrating high effectiveness.

### 6.6. Machine Learning

There are several NLP applications that employ the kNN classification algorithm, including text categorisation [37,38], question answering [39], text mining [40] and others. Other advanced applications of kNN include face recognition [41,42], emotion recognition [43,44], image recognition [45,46], handwriting recognition [47,48] and credit card fraud detection [49]. For example, finding similar words using word embeddings is a great example of kNN in high-dim space. Here, every document is considered as a vector. If the documents are close to each other, it means the documents contain identical topics or the documents are similar. Apart from that, it can also be used in time series [50], economic forecasting for predicting financial distress and many more applications [51].

## 7. Comparative Study

In this section, we have compared the state-of-the-art kNN Search and kNN Join approaches over high-dimensional datasets. This comparative study gives a better understanding of an approach and helps us figure out important features of the approach as well as its limitations.

### 7.1. kNN Search Techniques

We compared the various kNN Search approaches in Table 5 and we provided details on whether the considered exact approach is also further extended into approximate approaches to improve performance or not (i.e., both exact and approximate). In our analysis, we found that some studies mention they conducted experiments using high-dimensional datasets, but the datasets they used had dimensionalities such as 12D [117], 30D [112], 32D [109], etc. As a result, there is no industry standard for high dimensional values. Keeping the scenario in mind, we divide the dimensionality into three distinct ranges. Most existing techniques are classified in the first category. In the first range, we included everything from 2D to 99D. We included works in the second range that conducted experiments on datasets with dimensionalities ranging from 100D to 499D. In the third range, we consider the dataset to have a dimensionality equal to or greater than 500D.

In this section, we discussed the advantages and disadvantages of each of the techniques listed in Table 5.

**iDistance.** This method [110,111] makes it possible to obtain a small number of results right away while looking for more. That is, it gives a response to an online query, which is a key feature of interactive querying and data analysis. It is the most common baseline method used in kNN techniques and it has been tested with many different datasets. It outperforms various existing techniques as well, such as A-tree [187] and iMinMax [188], so we can say that it is robust and adaptive to different data distributions. A standard B${}^{+}$-tree is used to index the distance, as it only requires a very small amount of mapping effort. This makes it easy to integrate with a relational database management system.

The transformation of original *d*-dimensional datasets to 1D values is lossy. Thus, the chance of a false drop (i.e., an irrelevant or false data retrieval) happening is high during the iDistance search process. It cannot fix these false drops because some data points in high-dimensional space can be mapped to the same value in 1D space. It suffers from a bit high distance computation and cache misses because of its wide searching region. When the dimensionality of the iDistance technique becomes greater than 30D, the equidistant effect starts to happen, which makes pruning less effective quickly.

**Δ-tree.** They proposed a Δ-tree [74] as an effective new index technique for kNN Search. It helps optimise the computation of HD kNN queries in memory environments. It provides better pruning power, which means that it effectively cuts down the search space. This reduces the distance computation and speeds up the kNN Search in the main memory environment.

Its effectiveness depends on how well a dataset is globally correlated (i.e., works well for correlated data). To improve overall performance, the whole tree needs to be rebuilt every so often.

**array-index.** The array-index’s [117] compactness allows it to process the entire index structure in the main memory, which avoids disc activities. The ordering of partitions minimises the number of disc accesses required to obtain data pages and the ordering of data points inside partitions minimises the amount of computation required to determine the distances between the points in the retrieved data pages.

They do not consider the real-world dataset for experiments. It is seen that the synthetic data and real-world datasets provide different results. As we mostly deal with real-world datasets, the approach needs to be tested on real-world datasets.

**Diagonal Ordering.** The approach [112] can be used to facilitate an online query response, which involves providing an approximate query response by stopping the search process early. This is an outcome of the iterative searching method. We can use diagonal ordering to find a tight lower bound, which helps us get rid of irrelevant data points without having to do expensive distance calculations (i.e., providing better pruning power). The diagonal ordering index structure efficiently adjusts to various data distributions.

The experimental study was performed on a 30-dimensional dataset only, which does not guarantee that it will perform well over a very high-dimensional dataset.

**VA${}^{+}$-file.** Ref. [109] prevent excessively uneven data distribution across the clusters by restricting the cluster sizes from the top. This enhances the performance of a non-uniformly distributed dataset by using PCA and a non-uniform bit allocation.

To enable faster sequential scanning, the VA${}^{+}$-file offers approximate representations of data points. However, they have not taken into account the workload of the queries and the cache. The performance degrades as dimensionality increases. Basically, it uses the KLT, which is not scalable for large matrices.

**$\Delta^{+}$-tree.** In ref. [74,118], researchers use PCA to make it easier to create an index structure that supports pruning at various levels and with varying numbers of dimensions. It helps to minimise L2 cache misses and computational overhead. This technique also outperforms the iDistance, Pyramid-tree technique, etc.

The $\Delta^{+}$-tree also cannot stop a rebuilding process, but it can delay it for a sufficient amount of time. With varying datasets, the best suitable values of the parameters (such as clusters and segments) might also differ, because sometimes we are not aware of the distribution of the dataset.

**BF-CUDA.** Ref. [77] utilised the GPU to implement the fast, parallel kNN Search technique. It turns out that the NVIDIA CUDA API speeds up the kNN Search by 400 times the speed compared to using a brute force CPU-based approach.

This method cannot be used with very large datasets because it needs to calculate and store an entire *m* × *n* distance matrix.

**CUBLAS.** The compute unified device architecture (CUDA) and the CUDA basic linear algebra subprograms (CUBLAS) [75] provide the significant speedup, i.e., 25 times and 62 times faster on high-dimensional datasets, as compared to the highly optimised ANN C++ library [189].

It can be ineffective in large-scale environments. Actually, the brute force approach is often infeasible for big datasets as they have quadratic complexity. Utilising an index data structure may lower the quadratic complexity of brute force searches on the CPU or GPU. To find the kNN, we need to compare the query point with all the data points and select the k closest data points whose distances are the smallest. Even though GPGPUs are often used to speed up this method, the cost of moving data has a big effect on performance. However, utilising in-memory processing can greatly reduce the amount of data movement.

**ACDB.** They present an enhanced kNN Search method that uses adaptive cluster distance bounding [119] for indexing. It reduces CPU costs by removing unnecessary distance computations using triangle inequality.

The *k* value and the initial center pivots have a significant impact on the cluster technique. They perform an experiment considering that they have no impact on the cluster outcome. In this study, they utilised point-based triangular inequality to enhance kNN Searching, but it usually experiences memory overhead and inconsistent calculations, which leads to lesser performance.

**TBiS.** The TBiS [122] is beneficial in all the performance parameters; specifically, it offers synchronous concurrency, desired data locality and simple data and programme structures for an effective parallel kNN Search. The TBiS provides data independence. At each step, it divides the items evenly among the GPU’s threads so that there are no synchronisations or memory access conflicts.

TBiS needs additional transient global memory for intermediate computations. The study showed a sharp decline in performance as the number of data points *n* and closest neighbours *k* rose.

**QDBI.** The quad-tree-based distributed multidimensional data index (QBDI) [125] structure and the kNN query technique provide effective performance and scalability as compared to other P2P systems. It is not very focused on high-dimensional datasets. They limited the experiment to the dimensions 2D–5D.

In QDBI, the experiments are not performed on a real dataset. Considering most of the existing works discussed earlier, we can say that the synthetic data provides better results as compared to the real-world dataset because of several benefits of the synthetic datasets, such as data quality, scalability, etc. CUBLAS [75], for example, outperforms the ANN C++ library 189-fold on synthetic datasets and 62-fold on high-dimensional real-world datasets, according to experimental results. However, because these methods are meant to be used in the real world, they need to be tested with real-world, high-dimensional datasets.

**iDistance-PS.** Authors provide the first in-depth research on various partitioning techniques for the iDistance HD Indexing Technique. Ref. [120] helps us learn a lot more about why some methods work better than others when applied to datasets with different sizes, shapes and distributions. It helps find the best way to partition the datasets so that iDistance can have better pruning power. The new Partitioning Approaches provide efficient kNN querying for high-dimensional datasets. It also overcomes the limitations of the iDistance, such as the negative effect of points that are the same distance apart (equidistant) and partition overlap.

The problem of dimensionality is significantly worse with space-based approaches. By shifting the reference points, we can obtain better outcomes because the optimal location of reference points improves partitioning effectiveness.

**PL-tree.** To deal with the high-dimensional datasets, this new Indexing Technique [115] uses Cantor paring functions, which help scale well with dimensionality and data size. It outperforms the R*-tree and X-tree. It also supports efficient point queries, range queries and kNN queries.

It works better than the R*-tree and X-tree, but it is outperformed by the iDistance for point queries. They have done experiments with only 12 dimensions, but the real-world application creates data with hundreds or thousands of dimensions.

**CU-kNN.** GPUs with the parallel compute unified device architecture provide high performance and significant advantages. It helps address the core issues with CUDA-based data mining algorithms.

Ref. [121] only looks at how the techniques can be used on a single GPU. This makes them obsolete data mining methods, especially when you think about how well they work with big datasets.

**iDStar.** Ref. [162] explored a number of significant configurable factors to improve the performance of kNN queries utilising the iDistance and iDStar techniques, emphasising the challenges of indexing in HD and tightly clustered dataspaces.

It cannot filter the data points more effectively when clusters are a bit scattered. It also requires more time since more nodes must be visited. The problem of the curse of dimensionality still exists above the 256 dimensions.

**kNN-PA.** In [123], the authors provide methods that support closest neighbour searches over thousands of cores for arbitrary-dimensional datasets using the message passing interface and OpenMP.

The method is unable to perform well in a distributed memory environment because it is hard to predict how much memory will be used and there are a lot of communication costs. It was not designed for continuous updates, i.e., the insertion and deletion of data points.

**HC-O.** In ref. [116], the authors introduce the cache compact approximation representations of data points in main memory to speed up the candidate refining process during the kNN Search. It is a general method that can be used for both exact and approximate kNN Search methods.

They consider that the distribution of queries within the workload will not change quickly. The lower and upper bounds of query point *q* are calculated for every data point $p_{i}$ found in the cache. The authors then calculate the *k*-th minimum upper and lower bounds among all candidates. Data points whose $s_{i}.lb$ values are above $ub_{k}$ are pruned since they cannot be among the kNN. Additionally, they search for data points whose $s_{i}.ub$ is less than $lb_{k}$ since they could contain the needed solution and be added to the result set. That is why the effectiveness of this technique depends on the tightness of the distance bounds (and the histogram *H*).

**OTI and EOTI.** They provide a novel fast search technique based on the optimal triangle inequality-based (OTI) approach [113] for kNN and also provide an effective optimal triangle inequality-based check technique, taking into account the significant space complexity and additional time complexity of OTI. When searching for the *k* closest neighbours, the proposed triangle inequality-based techniques aid in reducing the costly redundant distance calculations.

The distances between each cluster centre and each given instance are computed and stored beforehand. The OTI’s space complexity for distance storage is S(N×M)=M×S(N), which can be considered unsatisfactory due to the high values of *N* and *M* in the huge datasets. Here, *N* is the number of instances in the dataset and *M* is the number of clusters in the dataset. The process of constructing triangles can be improved in order to increase the effectiveness of the kNN technique. As the flat-index buildings are built offline when the database is updated or the data changes, it is necessary to rebuild the data structures.

**BP.** In the BP method [114], using the PCCP helps to significantly lower the I/O cost and CPU time. The PCCP can minimise the number of data points, resulting in lower I/O costs and time consumption. Moreover, the I/O cost also lowers as the number of partitions rises and the CPU running time is minimised by setting the specified optimum value of partitions. These factors help improve CPU performance and reduce I/O costs. It is the first non-metric, high-dimensional technique that uses Bregman divergence to find the optimal kNN Search results.

By transforming Bregman distances into Euclidean distances and employing conventional metric searching methods, it is possible to solve the high-dimensional kNN Search problem more effectively. Moreover, modifying the BB-forest structure can facilitate massive data updates more effectively.

**HkNN.** The HkNN [124] offers a good hybrid, massively parallel closest neighbours approach that makes effective use of batching to distribute the computational burden between CPU and GPU. The presented hybrid technique performs linearly as data dimensionality increases. With high-dimensional datasets continuing to grow in size and dimension, high-performance closest neighbours search algorithms become very important.

They compared the results only with brute-force search techniques, as other tree-based approaches do not scale to very high-dimensional datasets (i.e., ≥ 500D), but they can be tested with high-dimensional datasets (i.e., 100D to 499D). There is scope to examine various optimised distance kernels, k selection approaches and combinations in distributed, hybrid and CPU environments.

### 7.2. kNN Join Techniques

Table 6 presents a comparative study of various kNN Join approaches discussed in this survey. Here we have divided the approaches into two partition strategies, i.e., space-based and data-based. The kNN Join techniques are classified based on the processing of the method. This Computing Paradigm taxonomy includes four different approaches: I/O, main memory, parallel and distributed. The taxonomy Indexing Techniques are discussed in Section 3 and Section 5. The Dimensionality Reduction Approach category includes various Dimensionality Reduction Approaches that play an important role in improving the capabilities of algorithms, such as reducing the distance computation cost.

In this section, we discussed the benefits and drawbacks of each of the techniques listed in Table 6.

**MuX.** The multipage index (MuX) [53,54] was used to effectively process the kNN Join. It employs page-loading and bucket-selection strategies to improve the performance of kNN. In order to decrease the CPU and I/O costs here, they have implemented the loading and processing strategies. The loading method accesses the hosting pages in decreasing order of quality. On the other hand, processing techniques solve the problem of loading the buckets of R and S into the cache in the right order so that they can be processed.

MuX employs an index to minimise the number of data pages that need to be accessed, but it still suffers since it uses an R-tree-based join technique. Similar to the R-tree, its performance is expected to decline as data dimensionality increases. It has a high memory overhead due to the space requirements of high-dimensional MBRs.

**Gorder.** This method [126] cuts down on CPU and I/O costs by sorting, scheduling joins, pruning and reducing the number of distance calculations. It essentially gains the benefit of a block-nested loop join in terms of minimising random access. Utilising a G-ordered data property, it removes from search any data blocks that are unfavourable in order to reduce I/O and similarity calculation costs. They used the distance computation reduction technique to further reduce the CPU cost, i.e., it prunes unnecessary block access. It manages high-dimensional data well while being simple and effective.

Gorder is a block-nested loop join technique where the pruning occurs at the block and object levels. Therefore, it requires a lot more computation. Every data point in the block keeps track of the *k* closest neighbours it has already visited, even though it computes the nearest neighbours of all points in a block before continuing to analyse data points in another block. With no reuse of neighbours of one point as neighbours of a point in close distance, each data point does this job individually and separately. It was created for static datasets and thus it is unable to work with dynamic datasets without having to recompute the whole kNN Join result.

**iJoin.** Applications and datasets supported by these approaches [128] are not limited. It can handle different *k* values because it is dynamic and adaptable.

MuX, Gorder and iJoin use the nested loops join technique, which needs to search a complete dataset. It needs to be recomputed to acquire the updated result if there is any change to either of the joining sets, i.e., the *R* or *S* dataset. None of these methods can be simply extended to process the incremental kNN Join.

**IIB and IIIB.** The threshold-based pruning [127] significantly lowers overhead, such as index creation and inverted list scanning. It also addresses the kNN Join problem for high-dimensional sparse datasets.

It was made for high-dimensional, sparse datasets, which can be ineffective for most correlated datasets. The effectiveness of the proposed algorithms can be optimised by using a more efficient refinement approach. They did not consider the existing state-of-the-art approaches for comparative study. The study only examined the brute force approach.

**kNNJoin${}^{+}$.** The kNNJoin${}^{+}$ method [76] searches for the *k* nearest neighbours efficiently and dynamically updates the results whenever an update operation is performed.

To manage the dynamic update, they present a kNNJoin${}^{+}$ technique based on the sphere-tree index. The sphere-tree follows an R-tree like structure, which normally has a mediocre performance in high-dimensional environments. Moreover, the high I/O cost makes it hard for disk-based solutions to meet real-time needs. They primarily focus on reducing I/O costs and face the issue of high node overlap and high computation costs of distances in high dimensional spaces.

**H-BNLJ and H-BRJ.** Ref. [130] proposes new ways to use MapReduce to do effective parallel kNN Joins on very large datasets.

Because the number of partitions is proportional to the number of blocks in each input dataset, the Hadoop block nested loop join (H-BNLJ) method does not work well with multidimensional or high-dimensional data. To perform parallel processing, Hadoop block R-tree join (H-BRJ) has to replicate dataset blocks. So, if we create *n* blocks, we have to make *n* copies of each block for a total of $n^{2}$ divisions, which causes a communication overhead.

**PGBJ.** This basically requires a low amount of disc space. In [58], the authors show that increasing the k value does not affect the communication overhead.

If the preprocessing and splitting steps do not reduce the number of search points by a large amount, the number of values that need to be sorted in the reduce step could be very high (up to |S|). The replication procedure requires computing several Euclidian distances (i.e., the cost of computing the distance in the preprocessing phase), making it computationally expensive. Therefore, it is not efficient for high-dimensional datasets. It shows a significant communication overhead. This is a result of the cell grouping and pivot selection. However, regardless of *k*, this overhead does not change.

**HDR-tree.** The researchers implement an HDR-tree [83] index structure to facilitate the effective search of affected users (i.e., users affected by the update operation). The proposed index structure provides an effective high-dimensional search by using clustering and PCA.

The HDR tree does not support optimised deletion. During the deletion operation, it has to recalculate the kNN for all query points, just like in static solutions. This is a costly operation and makes the process less efficient. It does not support batch updates. The performance is reduced because the kNN Join results must be updated every time a new item is added or updated in the dataset.

**EkNNJ.** In ref. [129], authors address the problem of existing kNN Join techniques, i.e., the lack of support for deletions and batch updates, with the help of new efficient deletion, lazy update and batch update algorithms.

It does not support a fully dynamic high-dimensional kNN Join, i.e., one static and one dynamic dataset where the user dataset is static while the item dataset is dynamic. To speed up the search process and deletion optimisation, authors maintained an RkNN table structure that continually mapped the item points in the sliding window to the corresponding reverse kNN list (RkNN). With the use of an RkNN table, they get the RkNN list of a deleted item instantly, but maintaining an RkNN table is a costly approach. Therefore, there is further scope for deletion optimisation.

## 8. Conclusions

We noticed that most researchers worked on approximation solutions to boost the effectiveness of the proposed techniques. However, there is a trade-off between efficiency and accuracy when using approximate strategies, which compromises precision to achieve efficiency. Several survey studies have been conducted on high-dimensional databases, but none have included exact kNN queries. Therefore, in this paper, we tried to cover almost all the high-dimensional kNN Search and kNN Join solutions. We provided a brief overview of all approaches and categorised these kNN query approaches based on five different factors, namely Indexing Technique, partitioning strategies, dimensionality reduction strategy, distance computation approach and Computing Paradigm. We included a comparison study as well in Section 7. In order to identify the limitations of the current approaches, we presented a comparative analysis of their merits and demerits. As far as we know, this will be the first detailed study of the exact kNN approaches over high-dimensional space.

## 9. Challenges and Future Directions

In order to address the problems of existing techniques, future research can focus on the following directions:

- In the approaches that use a cluster-based partitioning strategy, finding the ideal number of clusters is always difficult because it mostly depends on the size of the dataset, the number of features and the distribution of the data points. So, we can take into account all these factors when designing an efficient approach. It will help to improve the performance.
- A clustering approach is used for partitioning, which provides indexes with better pruning power. So, considering the importance of effective search in high-dimensional space, research can be conducted on the quality of dataset clustering to distribute the data instances within clusters more reasonably.
- The structure of the relevant triangle has a big effect on how well a search method based on triangle inequality works. As a result, existing works use an efficient optimal triangle-inequality approach to select an optimal cluster centre from among all cluster centres that aid in the construction of a suitable triangle. In order to reduce space and time complexity further, the triangle’s composition can be optimised by creating a few promising reference points and using them to make optimum triangles.
- The analysis in Table 3 and Table 4 shows that most approaches do not support dynamic datasets. Since real-world applications are increasingly using kNN queries, future work needs to focus more on dynamic datasets.
- As per our survey, not a single parallel and distributed exact kNN Join approach for a high-dimensional dataset is available. Distributed parallel computing offers several problem-solving capabilities. Using it as a direction for research can improve the overall performance of kNN queries over high-dimensional data by a large amount. The approaches that are available support up to 20 dimensionalities only. So, there is room for further research in kNN Join over high-dimensional data based on parallel, distributed or hybrid Computing Paradigms.

## Figures and Tables

**Table 2:** Summary of commonly used symbol and definitions.

Symbols	Definitions
R,S	Datasets
$r_{i},s_{j}$	Data points of R and S dataset
*k*	number of nearest neighbours
d(x,y)	Euclidean distance function
d,D	Dimensionality of original dataset
*q*	Query data point
$R^{d}$	*d*-dimensional space

**Table 3:** Summary of kNN Search Techniques.

Comp. Para.	Part ${}^{n}$ Strat.	Indexing Technique	Techniques	Dim. Reduction Approach	App.	Synth. Dim.	Real. Dim.	Dyn. Data	Dist. Mt.	Time. Comp.
I/O	Space	VA${}^{+}$-file	VA${}^{+}$-file [109]	KLT	Both	N/A	Mod.	No	L2	O(log n) *
	Data	B${}^{+}$-tree	iDistance [110,111]	iDistance	Exact	Mod.	Mod.	Yes	L2	O(log n) *
			Diagonal Ordering [112]	PCA	Exact	Mod.	Mod.	No	L2	O(log n) *
		N/A	OTI & EOTI [113]	N/A	Exact	N/A	High+	No	L2	O(n)
		BB-trees	BP [114]	PCCP	Both	High	High	No	Breg. dist.	O(nlogn) *
MEM.	Space	R-tree	PL-Tree [115]	CPF	Exact	Mod.	Mod.	Yes	L2	O(log n) *
		Hash-based	HC-O [116]	N/A	Both	N/A	High+	No	L2	O(n)
	Data	Δ-tree	Δ-tree [74]	PCA	Exact	Moerate	Mod.	Yes	L2	O(n) *
		1D array	Array-index [117]	array-index	Exact	Mod.	N/A	No	L2	O(log n) *
		$\Delta^{+}$-tree	$\Delta^{+}$-tree [74,118]	PCA	Exact	Mod.	Mod.	Yes	L2	O(n) *
		ACDB	ACDB [119]	N/A	Exact	N/A	Mod.	No	L2	O(nlogn) *
		B${}^{+}$-tree	iDistance-PS [120]	iDistance	Exact	Mod.	High	No	L2	O(n) *
			iDStar [115]	iDistance	Exact	High+	High	No	L2	O(n) *
PARL.	Space	N/A	CU-kNN [121]	1D reduct${}^{n}$	Exact	Mod.	Mod.	No	L2	O(n) *
			TBiS [122]	N/A	Exact	N/A	High	No	L2	O(log${}^{2}$ n)
		Randomised k dim. tree	kNN-PA [123]	N/A	Both	High+	High+	No	L2	O(nlogn) *
	Data	N/A	HkNN [124]	N/A	Exact	High+	N/A	No	L2	O(n) *
	No Part ${}^{n}$	N/A	BF-CUDA [77]	N/A	Exact	Mod.	Mod.	No	L2	O(n) *
			CUBLAS [75]	N/A	Exact	High	High	No	L2	O(log n) *
DISTR.	Space	Quad-trees	QDBI [125]	N/A	Exact	Mod.	N/A	No	L2	O(log n) *

Comp Para, computing paradigm; Part ^*n*^ Strat, partition strategy; Dim Reduct ^*n*^ App, dimensionality reduction approach; Synth Dim, synthetic datasets dimensionality; Real Dim, real-world datasets dimensionality; Dyn Data, support dynamic datasets; Dist Mt, distance metric; Time Comp, time complexity; Space, space-based; Data, data-based; No Part ^*n*^, no partition; PCA, Principal Component Analysis; KLT, Karhunen Loève Transform; PCCP, Pearson Correlation Coefficient-based Partition; CPF, cantor pairing function; Both, exact and approximate; Mod, moderate dimensionality (2D–99D); High, high dimensionality (100D–499D); High+, very high dimensionality (500D and above); N/A, not applicable(does not use any); No Partition, do not use any partition technique; L2, Euclidean distance, *, time complexity is not informed by the authors.

**Table 4:** Summary of kNN Join Techniques.

Comp. Para.	Part ${}^{n}$ Strat.	Indexing Technique	Techniques	Dim. Reduct ${}^{n}$ App.	App.	Synth. Dim.	Real. Dim.	Dyn. Data	Dist. Mt.	Time Comp.
I/O	Space	R-Tree	MuX [53,54]	N/A	Exact	Mod.	Mod.	No	L2	O(nlogn)
		N/A	Gorder [126]	PCA	Exact	Mod.	Mod.	No	L2	O(n${}^{2}$) *
		inverted index	IIB & IIIB [127]	N/A	Exact	High+	Mod.	No	L2	O(nlogn) *
	Data	iDistance	iJoin [128]	PCA	Exact	Mod.	Mod.	No	L2	O(nlogn) *
			kNNJoin${}^{+}$ [76]	iDistance	Exact	Mod.	Mod.	Yes	L2	O(nlogn) *
MEM.	Data	HDR-Tree	HDR-Tree [83]	PCA	Both	High	High	Yes	L2	O(nlogn)
			EkNN [129]	PCA	Exact	N/A	High+	Yes	L2	O(nlogn) *
DISTR. & PARL.	Space	R-Tree	H-BNLJ & H-BRJ [130]	N/A	Both	Mod.	Mod.	No	L2	O(n${}^{2}$) *
	Data	N/A	PGBJ [58]	N/A	Both	Mod.	Mod.	No	L2	O(nlogn) *

Comp Para, computing paradigm; Part ^*n*^ Strat, partition strategy; Dim Reduct ^*n*^ App, dimensionality reduction approach; Synth Dim, synthetic datasets dimensionality; Real Dim, real-world datasets dimensionality; Dyn Data, support dynamic datasets; Dist Mt, distance metric; Time Comp, time complexity; Space, space-based; Data, data-based; PCA, Principal Component Analysis; Both, exact and approximate; Mod, moderate dimensionality (2D–99D); High, high dimensionality (100D–499D); High+, very high dimensionality (500D and above); N/A, not applicable (does not use any); No Partition, do not use any partition technique; L2, Euclidean distance, *, time complexity is not informed by the authors.

**Table 5:** Comparative Study of kNN Search.

Sr No.	Techniques	Merits	Demerits
1.	iDistance [110,111]	1. Support online query answering 2. Robust and adaptive to different data distributions 3. It can be integrated into DBMS cost-effectively	1. It has a wide search region 2. Lossy transformation leads to false drops 3. When dim. increases, pruning efficiency decreases
2.	Δ-tree [74]	1. Provides an optimised index structure 2. Reduce the search space and speed up the kNN query in the main memory environment	1. Effective for correlated dataset 2. Entire tree must be rebuilt on a recurring basis
3.	array-index [117]	1. Minimal disk access 2. It is simple and compact, yet faster	1. Not considered real-life dataset for experiments
4.	Diagonal Ordering [112]	1. It avoids unnecessary distance computation 2. It can effectively adapt to varied data distributions 3. Supports online query answering	1. Experimental study was performed on a 30D dataset only, which doesn’t guarantee to outperform a very high-dimensional dataset
5.	VA${}^{+}$-file [109]	1. Prevents excessively uneven data distribution across the clusters 2. Useful for kNN Search in non-uniform datasets	1. For better search results, consider approximation 2. The performance degrades as dim. increases 3. Do not consider the cache and query workload
6.	$\Delta^{+}$-tree [74,118]	1. It helps to minimise the computational cost and cache misses 2. Outperforms iDistance, Pyramid tree, etc.	1. It cannot stop a tree-rebuilding process 2. With varying datasets, the optimum values of parameters (like clusters) might also differ
7.	BF-CUDA [77]	1. Supports fast, parallel kNN Search	1. Unable to scale to a very large dataset
8.	CUBLAS [75]	1. It provides a significant speedup and outperforms the ANN C++ library	1. The performance gets impacted by the cost of data movement 2. It can be ineffective for large-scale environments
9.	ACDB [119]	1. Using the triangle inequality, CPU cost was effectively minimised and provided better performance	1. The initial center pivots and *k* value both have a significant impact on the cluster method
10.	TBiS [122]	1. Offers simple data and programme structures, synchronous concurrency and optimal data localisation	1. With an increasing number of data items and *k* value, the performance dramatically degrades
11.	QDBI [125]	1. Good scalability and search performance	1. Experiments not performed on a real dataset 2. Not much focused on high-dimensional dataset
12.	iDistance-PS [120]	1. Enhancements to the filtering power of iDistance 2. Provides efficient kNN querying	1. The problem of dimensionality is significantly worse in space-based approaches
13.	PL-Tree [115]	1. It can scale well with dimensionality and data size. Also supports efficient point queries & range queries	1. Perform experiments using 12D dim. only 2. iDistance outperforms PL-Tree for point queries
14.	CU-kNN [121]	1. Provides high-performance 2. It improves core issues with CUDA-based data mining algorithms	1. Suffers from big data scalability 2. Data movement and the cost is an issue
15.	iDStar [115]	1. It performs better in high-dimensional, tightly clustered dataspaces	1. Less pruning power for scattered clusters 2. Not much effective for dimensionality above 256
16.	kNN-PA [123]	1. It is scalable and allows kNN Searches for any HD datasets over thousands of cores	1. Not designed for continuous point updates 2. Ineffective in a distributed-memory environment
17.	HC-O [116]	1. It speeds up the candidate refining process during the kNN Search 2. It is a fundamental method that works for both exact and approximate kNN Search techniques	1. In the workload, they presume a stable distribution of queries 2. The tightness of the distance bounds and the histogram affects the pruning power
18.	OTI and EOTI [113]	1. Lower complexity and faster search algorithm 2. It reduces costly distance computation	1. OTI suffers from large space and time complexity 2. The process of constructing triangles can be improved for better efficiency
19.	BP [114]	1. It is the first high-dimensional non-metric Bregman divergence that provides a better kNN Search 2. Performance improvements in CPU time & IO cost	1. Does not effectively support massive data updates 2. By transforming Bregman distance to L2, more effective solutions can be implemented
20.	HkNN [124]	1. Effectively split work between the CPU and GPU 2. It provides a hybrid approach for the kNN Search 3. Effectiveness grows linearly with increasing dim.	1. Compared results using only brute-force tech. 2. There is scope to examine various optimised k selection approaches and distance kernels

**Table 6:** Comparative Study of kNN Join Techniques.

Sr No.	Techniques	Merits	Demerits
1.	MuX [53,54]	1. Designed to reduce the I/O and CPU costs	1. Performance degrades with an increased dim. 2. High memory overhead
2.	Gorder [126]	1. Reduce random access 2. Prunes the unpromising block blocks	1. It requires significantly more computation 2. Designed for static data
3.	iJoin [128]	1. Adaptive and dynamic	1. Very costly for dynamic data
4.	IIB and IIIB [127]	1. Effective for the sparse dataset	1. It may not be feasible for correlated datasets. Algorithms’ effectiveness can be optimised
5.	kNNJoin${}^{+}$ [76]	1. It supports efficient searching and dynamic updates	1. Unable to meet the real-time requirements 2. High distance computation cost & node overlap
6.	H-BNLJ and H-BRJ [130]	1. Easy to implement	1. Slower and unable to scale well 2. Communication overhead is very high
7.	PGBJ [58]	1. Increasing the *k* value does not affect the communication overhead 2. Disk usage is very low	1. Inefficient for high-dimensional data 2. Pivot selection significantly affects performance 3. time-consuming operations for big datasets
8.	HDR-tree [83]	1. Searches for affected users are made efficient 2. It is effective for high-dimensional data	1. Lack of Support for Deletions 2. Lack of Support for Batch Updates
9.	EkNNJ [129]	1. Efficient dynamic update, batch and lazy update 2. Provide optimised deletion	1. Does not support fully dynamic HD kNN Join 2. Scope for deletion optimisation

## Data Availability

Not applicable.

## References

[1] Andoni A., Indyk P. (2008). Near-Optimal hashing algorithms for approximate nearest neighbour in high dimensions. Commun. ACM.

[2] Bawa M., Condie T., Ganesan P. LSH forest: Self-tuning indexes for similarity search. Proceedings of the 14th international conference on World Wide Web.

[3] Lv Q., Josephson W., Wang Z., Charikar M., Li K. Multi-probe LSH: Efficient indexing for high-dimensional similarity search. Proceedings of the 33rd International Conference on Very Large Data Bases.

[4] Jegou H., Douze M., Schmid C. (2010). Product quantization for nearest neighbour search. IEEE Trans. Pattern Anal. Mach. Intell..

[5] Wang Y., Pan Z., Li R. (2019). A new cell-level search based non-exhaustive approximate nearest neighbour (ANN) search algorithm in the framework of product quantization. IEEE Access.

[6] Li W., Yi P., Wu Y., Pan L., Li J. (2014). A new intrusion detection system based on kNN classification algorithm in wireless sensor network. J. Electr. Comput. Eng..

[7] Liu G., Zhao H., Fan F., Liu G., Xu Q., Nazir S. (2022). An Enhanced Intrusion Detection Model Based on Improved kNN in WSNs. Sensors.

[8] Yang J., Sun Z., Chen Y. (2016). Fault detection using the clustering-kNN rule for gas sensor arrays. Sensors.

[9] Wang G.Z., Li J., Hu Y.T., Li Y., Du Z.Y. (2019). Fault identification of chemical processes based on k NN variable contribution and CNN data reconstruction methods. Sensors.

[10] Zhou C., Tham C.K. Graphel: A graph-based ensemble learning method for distributed diagnostics and prognostics in the industrial internet of things. Proceedings of the 2018 IEEE 24th International Conference on Parallel and Distributed Systems (ICPADS).

[11] Liang S., Ning Y., Li H., Wang L., Mei Z., Ma Y., Zhao G. (2015). Feature selection and predictors of falls with foot force sensors using kNN-based algorithms. Sensors.

[12] Dziubany M., Machhamer R., Laux H., Schmeink A., Gollmer K.U., Burger G., Dartmann G. Machine learning based indoor localization using a representative k nearest-neighbour classifier on a low-cost IoT-hardware. Proceedings of the 2018 26th European Signal Processing Conference (EUSIPCO).

[13] Ferreira D., Souza R., Carvalho C. (2020). Qa-knn: Indoor localization based on quartile analysis and the knn classifier for wireless networks. Sensors.

[14] Al-Faiz M.Z., Ali A.A., Miry A.H. A k nearest neighbour based algorithm for human arm movements recognition using EMG signals. Proceedings of the 2010 1st International Conference on Energy, Power and Control (EPC-IQ).

[15] Shen B., Zhao Y., Li G., Zheng W., Qin Y., Yuan B., Rao Y. V-tree: Efficient kNN Search on moving objects with road-network constraints. Proceedings of the 2017 IEEE 33rd International Conference on Data Engineering (ICDE).

[16] Fiorini L., Mancioppi G., Semeraro F., Fujita H., Cavallo F. (2020). Unsupervised emotional state classification through physiological parameters for social robotics applications. Knowl.-Based Syst..

[17] Markom M., Adom A., Shukor S.A., Rahim N.A., Tan E.M.M., Ilias B. (2019). Improved kNN Scan Matching for Local Map Classification in Mobile Robot Localisation Application. Proceedings of the IOP Conference Series: Materials Science and Engineering.

[18] Pinto A.M., Rocha L.F., Moreira A.P. (2013). Object recognition using laser range finder and machine learning techniques. Robot. Comput.-Integr. Manuf..

[19] Xu G., Pang Y., Bai Z., Wang Y., Lu Z. (2021). A fast point clouds registration algorithm for laser scanners. Appl. Sci..

[20] Zheng B., Zheng K., Xiao X., Su H., Yin H., Zhou X., Li G. Keyword-aware continuous knn query on road networks. Proceedings of the 2016 IEEE 32Nd international conference on data engineering (ICDE).

[21] Tripathy D., Parida S., Khandu L. (2021). Safety risk assessment and risk prediction in underground coal mines using machine learning techniques. J. Inst. Eng. (India) Ser. D.

[22] Mohsen S., Elkaseer A., Scholz S.G. Human activity recognition using K-nearest neighbour machine learning algorithm. Proceedings of the International Conference on Sustainable Design and Manufacturing.

[23] Patro S.G.K., Mishra B.K., Panda S.K., Kumar R., Long H.V., Taniar D., Priyadarshini I. (2020). A hybrid action-related K-nearest neighbour (HAR-kNN) approach for recommendation systems. IEEE Access.

[24] Subramaniyaswamy V., Logesh R. (2017). Adaptive kNN based recommender system through mining of user preferences. Wirel. Pers. Commun..

[25] Li G., Zhang J. Music personalized recommendation system based on improved kNN algorithm. Proceedings of the 2018 IEEE 3rd Advanced Information Technology, Electronic and Automation Control Conference (IAEAC).

[26] Cover T., Hart P. (1967). Nearest neighbour pattern classification. IEEE Trans. Inf. Theory.

[27] Pan Z., Wang Y., Ku W. (2017). A new k harmonic nearest neighbour classifier based on the multi-local means. Expert Syst. Appl..

[28] Pan Z., Wang Y., Ku W. (2017). A new general nearest neighbour classification based on the mutual neighbourhood information. Knowl.-Based Syst..

[29] De Figueiredo J., Oliveira F., Esmi E., Freitas L., Schleicher J., Novais A., Sussner P., Green S. (2013). Automatic detection and imaging of diffraction points using pattern recognition. Geophys. Prospect..

[30] Nguyen B., Morell C., De Baets B. (2016). Large-scale Distance Metric learning for k nearest neighbours regression. Neurocomputing.

[31] Song Y., Liang J., Lu J., Zhao X. (2017). An efficient instance selection algorithm for k nearest neighbour regression. Neurocomputing.

[32] Stone C.J. (1977). Consistent nonparametric regression. Ann. Stat..

[33] Angiulli F., Basta S., Pizzuti C. (2005). Distance-based detection and prediction of outliers. IEEE Trans. Knowl. Data Eng..

[34] Ghoting A., Parthasarathy S., Otey M.E. (2008). Fast mining of distance-based outliers in high-dimensional datasets. Data Min. Knowl. Discov..

[35] Ning J., Chen L., Zhou C., Wen Y. (2018). Parameter k search strategy in outlier detection. Pattern Recognit. Lett..

[36] Ramaswamy S., Rastogi R., Shim K. Efficient algorithms for mining outliers from large datasets. Proceedings of the 2000 ACM SIGMOD International Conference on Management of Data.

[37] Li B., Yu S., Lu Q. (2003). An improved k nearest neighbour algorithm for text categorization. arXiv.

[38] Jiang S., Pang G., Wu M., Kuang L. (2012). An improved K-nearest-neighbour algorithm for text categorization. Expert Syst. Appl..

[39] Soares J.N., Cavalcante H.G., Maia J.E. (2021). A Question Classification in Closed Domain Question-Answer Systems. Int. J. Appl. Inf. Syst..

[40] Bijalwan V., Kumar V., Kumari P., Pascual J. (2014). kNN based machine learning approach for text and document mining. Int. J. Database Theory Appl..

[41] Zhao J., Han J., Shao L. (2017). Unconstrained face recognition using a set-to-set distance measure on deep learned features. IEEE Trans. Circuits Syst. Video Technol..

[42] Tofighi A., Khairdoost N., Monadjemi S.A., Jamshidi K. (2014). A robust face recognition system in image and video. Int. J. Image, Graph. Signal Process..

[43] Zhang J., Yin Z., Chen P., Nichele S. (2020). Emotion recognition using multi-modal data and machine learning techniques: A tutorial and review. Inf. Fusion.

[44] Murugappan M. Human emotion classification using wavelet transform and kNN. Proceedings of the 2011 International Conference on Pattern Analysis and Intelligence Robotics.

[45] Guru D., Sharath Y., Manjunath S. (2010). Texture features and kNN in classification of flower images. IJCA Spec. Issue RTIPPR (1).

[46] Zawbaa H.M., Abbass M., Hazman M., Hassenian A.E. Automatic fruit image recognition system based on shape and color features. Proceedings of the International Conference on Advanced Machine Learning Technologies and Applications.

[47] Zanchettin C., Bezerra B.L.D., Azevedo W.W. A kNN-SVM hybrid model for cursive handwriting recognition. Proceedings of the 2012 International Joint Conference on Neural Networks (IJCNN).

[48] Hamid N.A., Sjarif N.N.A. (2017). Handwritten recognition using SVM, kNN and neural network. arXiv.

[49] Akila S., Reddy U.S. (2018). Cost-sensitive Risk Induced Bayesian Inference Bagging (RIBIB) for credit card fraud detection. J. Comput. Sci..

[50] Lytridis C., Lekova A., Bazinas C., Manios M., Kaburlasos V.G. (2020). WINkNN: Windowed intervals’ number kNN classifier for efficient time-series applications. Mathematics.

[51] Imandoust S.B., Bolandraftar M. (2013). Application of k nearest neighbour (knn) approach for predicting economic events: Theoretical background. Int. J. Eng. Res. Appl..

[52] Knuth D.E. (1973). The Art of Computer Programming.

[53] Böhm C., Krebs F. Supporting KDD applications by the k nearest neighbour join. Proceedings of the International Conference on Database and Expert Systems Applications.

[54] Böhm C., Krebs F. (2004). The k nearest neighbour join: Turbo charging the kdd process. Knowl. Inf. Syst..

[55] Hartigan J.A., Wong M.A. (1979). Algorithm AS 136: A k means clustering algorithm. J. R. Stat. Society. Ser. C (Appl. Stat.).

[56] Kanungo T., Mount D.M., Netanyahu N.S., Piatko C.D., Silverman R., Wu A.Y. (2002). An efficient k means clustering algorithm: Analysis and implementation. IEEE Trans. Pattern Anal. Mach. Intell..

[57] Breunig M.M., Kriegel H.P., Ng R.T., Sander J. LOF: Identifying density-based local outliers. Proceedings of the 2000 ACM SIGMOD International Conference on Management of Data.

[58] Lu W., Shen Y., Chen S., Ooi B.C. (2012). Efficient processing of k nearest neighbour joins using mapreduce. arXiv.

[59] Dasarathy B.V. (1991). Nearest neighbour (NN) norms: NN pattern classification techniques. IEEE Comput. Soc. Tutor..

[60] Zhang S., Li X., Zong M., Zhu X., Wang R. (2017). Efficient kNN classification with different numbers of nearest neighbours. IEEE Trans. Neural Networks Learn. Syst..

[61] Guttman A. R-trees: A dynamic index structure for spatial searching. Proceedings of the 1984 ACM SIGMOD International Conference on Management of Data.

[62] Beckmann N., Kriegel H.P., Schneider R., Seeger B. The R*-tree: An efficient and robust access method for points and rectangles. Proceedings of the 1990 ACM SIGMOD International Conference on Management of Data.

[63] Kamel I., Faloutsos C. Hilbert R-tree: An Improved R-Tree Using Fractals. Proceedings of the VLDB’94: Proceedings of the 20th International Conference on Very Large Data Bases.

[64] Arge L., Berg M.d., Haverkort H., Yi K. (2008). The priority R-tree: A practically efficient and worst-case optimal R-tree. ACM Trans. Algorithms (TALG).

[65] Sproull R.F. (1991). Refinements to nearest-neighbour searching ink-dimensional trees. Algorithmica.

[66] Fukunaga K., Narendra P.M. (1975). A branch and bound algorithm for computing k nearest neighbours. IEEE Trans. Comput..

[67] Yianilos P.N. Data structures and algorithms for nearest neighbour. Proceedings of the fourth annual ACM-SIAM Symposium on Discrete algorithms.

[68] Bozkaya T., Ozsoyoglu M. Distance-based indexing for high-dimensional metric spaces. Proceedings of the 1997 ACM SIGMOD International Conference on Management of Data.

[69] Li Y., Guo L. (2007). An active learning based TCM-kNN algorithm for supervised network intrusion detection. Comput. Secur..

[70] Shapoorifard H., Shamsinejad P. (2017). Intrusion detection using a novel hybrid method incorporating an improved kNN. Int. J. Comput. Appl.

[71] Weber R., Schek H.J., Blott S. (1998). A quantitative analysis and performance study for similarity-search methods in high-dimensional spaces. Proc. VLDB.

[72] Beyer K., Goldstein J., Ramakrishnan R., Shaft U. When is “nearest neighbour” meaningful?. Proceedings of the International Conference on Database Theory.

[73] Kouiroukidis N., Evangelidis G. The effects of dimensionality curse in high dimensional kNN Search. Proceedings of the 2011 15th Panhellenic Conference on Informatics.

[74] Cui B., Ooi B.C., Su J., Tan K.L. Contorting high dimensional data for efficient main memory kNN processing. Proceedings of the 2003 ACM SIGMOD International Conference on Management of Data.

[75] Garcia V., Debreuve E., Nielsen F., Barlaud M. K-nearest neighbour search: Fast GPU-based implementations and application to high-dimensional feature matching. Proceedings of the 2010 IEEE International Conference on Image Processing.

[76] Yu C., Zhang R., Huang Y., Xiong H. (2010). High-dimensional kNN Joins with incremental updates. Geoinformatica.

[77] Garcia V., Debreuve E., Barlaud M. Fast k nearest neighbour search using GPU. Proceedings of the 2008 IEEE Computer Society Conference on Computer Vision and Pattern Recognition Workshops.

[78] Wold S., Esbensen K., Geladi P. (1987). Principal component analysis. Chemom. Intell. Lab. Syst..

[79] Chakrabarti K., Mehrotra S. Local dimensionality reduction: A new approach to indexing high dimensional spaces. Proceedings of the VLDB Conference.

[80] Abdi H., Williams L.J. (2010). Principal component analysis. Wiley Interdiscip. Rev. Comput. Stat..

[81] Vidal R., Ma Y., Sastry S.S. (2016). Principal component analysis. Generalized Principal Component Analysis.

[82] Ciaccia P., Patella M., Zezula P. (1997). M-tree: An efficient access method for similarity search in metric spaces. Proc. Vldb.

[83] Yang C., Yu X., Liu Y. Continuous kNN Join processing for real-time recommendation. Proceedings of the 2014 IEEE International Conference on Data Mining.

[84] Kibriya A.M., Frank E. An empirical comparison of exact nearest neighbour algorithms. Proceedings of the European Conference on Principles of Data Mining and Knowledge Discovery.

[85] Bhatia N. (2010). Survey of nearest neighbour techniques. arXiv.

[86] Abbasifard M.R., Ghahremani B., Naderi H. (2014). A survey on nearest neighbour search methods. Int. J. Comput. Appl..

[87] Liu T., Moore A., Yang K., Gray A. (2004). An investigation of practical approximate nearest neighbour algorithms. Adv. Neural Inf. Process. Syst..

[88] Li W., Zhang Y., Sun Y., Wang W., Li M., Zhang W., Lin X. (2019). Approximate nearest neighbour search on high dimensional data—experiments, analyses and improvement. IEEE Trans. Knowl. Data Eng..

[89] Song G., Rochas J., Huet F., Magoules F. Solutions for processing k nearest neighbour joins for massive data on mapreduce. Proceedings of the 2015 23rd Euromicro International Conference on Parallel, Distributed and Network-Based Processing.

[90] Song G., Rochas J., El Beze L., Huet F., Magoules F. (2016). K nearest neighbour joins for big data on mapreduce: A theoretical and experimental analysis. IEEE Trans. Knowl. Data Eng..

[91] Adomavicius G., Tuzhilin A. (2005). Toward the next generation of recommender systems: A survey of the state-of-the-art and possible extensions. IEEE Trans. Knowl. Data Eng..

[92] Boiman O., Shechtman E., Irani M. In defense of nearest-neighbour based image classification. Proceedings of the 2008 IEEE Conference on Computer Vision and Pattern Recognition.

[93] Malkov Y., Ponomarenko A., Logvinov A., Krylov V. (2014). Approximate nearest neighbour algorithm based on navigable small world graphs. Inf. Syst..

[94] Iwasaki M. Pruned bi-directed k nearest neighbour graph for proximity search. Proceedings of the International Conference on Similarity Search and Applications.

[95] Malkov Y.A., Yashunin D.A. (2018). Efficient and robust approximate nearest neighbour search using hierarchical navigable small world graphs. IEEE Trans. Pattern Anal. Mach. Intell..

[96] Hajebi K., Abbasi-Yadkori Y., Shahbazi H., Zhang H. Fast approximate nearest-neighbour search with k nearest neighbour graph. Proceedings of the Twenty-Second International Joint Conference on Artificial Intelligence.

[97] Zhang Y.M., Huang K., Geng G., Liu C.L. Fast kNN graph construction with locality sensitive hashing. Proceedings of the Joint European Conference on Machine Learning and Knowledge Discovery in Databases.

[98] Zhao W.L., Yang J., Deng C.H. (2017). Scalable nearest neighbour search based on kNN graph. arXiv.

[99] Yang J., Zhao W.L., Deng C.H., Wang H., Moon S. Fast nearest neighbour search based on approximate k NN graph. Proceedings of the International Conference on Internet Multimedia Computing and Service.

[100] Alshammari M., Stavrakakis J., Takatsuka M. (2021). Refining a k nearest neighbour graph for a computationally efficient spectral clustering. Pattern Recognit..

[101] Fu C., Xiang C., Wang C., Cai D. (2017). Fast approximate nearest neighbour search with the navigating spreading-out graph. arXiv.

[102] Fu C., Cai D. (2016). Efanna: An extremely fast approximate nearest neighbour search algorithm based on knn graph. arXiv.

[103] Harwood B., Drummond T. Fanng: Fast approximate nearest neighbour graphs. Proceedings of the IEEE Conference on Computer Vision and Pattern Recognition.

[104] Munoz J.V., Gonçalves M.A., Dias Z., Torres R.d.S. (2019). Hierarchical clustering-based graphs for large scale approximate nearest neighbour search. Pattern Recognit..

[105] Fu C., Wang C., Cai D. (2021). High dimensional similarity search with satellite system graph: Efficiency, scalability and unindexed query compatibility. IEEE Trans. Pattern Anal. Mach. Intell..

[106] Aumüller M., Bernhardsson E., Faithfull A. (2020). ANN-Benchmarks: A benchmarking tool for approximate nearest neighbour algorithms. Inf. Syst..

[107] Shimomura L.C., Oyamada R.S., Vieira M.R., Kaster D.S. (2021). A survey on graph-based methods for similarity searches in metric spaces. Inf. Syst..

[108] Wang M., Xu X., Yue Q., Wang Y. (2021). A comprehensive survey and experimental comparison of graph-based approximate nearest neighbour search. arXiv.

[109] Ferhatosmanoglu H., Tuncel E., Agrawal D., El Abbadi A. (2006). High dimensional nearest neighbour searching. Inf. Syst..

[110] Yu C., Ooi B.C., Tan K.L., Jagadish H. (2001). Indexing the distance: An efficient method to knn processing. Proc. Vldb.

[111] Jagadish H.V., Ooi B.C., Tan K.L., Yu C., Zhang R. (2005). iDistance: An adaptive B+-tree based indexing method for nearest neighbour search. ACM Trans. Database Syst. (TODS).

[112] Hu J., Cui B., Shen H. Diagonal ordering: A new approach to high-dimensional kNN processing. Proceedings of the 15th Australasian database conference.

[113] Pan Y., Pan Z., Wang Y., Wang W. (2020). A new fast search algorithm for exact k nearest neighbours based on optimal triangle-inequality-based check strategy. Knowl.-Based Syst..

[114] Song Y., Gu Y., Zhang R., Yu G. (2020). Brepartition: Optimized high-dimensional kNN Search with bregman distances. IEEE Trans. Knowl. Data Eng..

[115] Wang J., Lu J., Fang Z., Ge T., Chen C. PL-Tree: An efficient indexing method for high-dimensional data. Proceedings of the International Symposium on Spatial and Temporal Databases.

[116] Tang B., Yiu M.L., Hua K.A. (2016). Exploit every bit: Effective caching for high-dimensional nearest neighbour search. IEEE Trans. Knowl. Data Eng..

[117] Al Aghbari Z., Makinouchi A. Linearization approach for efficient kNN Search of high-dimensional data. Proceedings of the International Conference on Web-Age Information Management.

[118] Cui B., Coi B.C., Su J., Tan K.L. (2005). Indexing high-dimensional data for efficient in-memory similarity search. IEEE Trans. Knowl. Data Eng..

[119] Hong H., Juan G., Ben W. An improved kNN algorithm based on adaptive cluster distance bounding for high dimensional indexing. Proceedings of the 2012 Third Global Congress on Intelligent Systems.

[120] Schuh M.A., Wylie T., Banda J.M., Angryk R.A. A comprehensive study of idistance partitioning strategies for knn queries and high-dimensional data indexing. Proceedings of the British National Conference on Databases.

[121] Jian L., Wang C., Liu Y., Liang S., Yi W., Shi Y. (2013). Parallel data mining techniques on graphics processing unit with compute unified device architecture (CUDA). J. Supercomput..

[122] Sismanis N., Pitsianis N., Sun X. Parallel search of k nearest neighbours with synchronous operations. Proceedings of the 2012 IEEE Conference on High Performance Extreme Computing.

[123] Xiao B., Biros G. (2016). Parallel algorithms for nearest neighbour search problems in high dimensions. SIAM J. Sci. Comput..

[124] Muhr D., Affenzeller M. Hybrid (CPU/GPU) Exact Nearest Neighbours Search in High-Dimensional Spaces. Proceedings of the IFIP International Conference on Artificial Intelligence Applications and Innovations.

[125] Qiao B., Ding L., Wei Y., Wang X. A kNN Query Processing Algorithm over High-Dimensional Data Objects in P2P Systems. Proceedings of the 2011 2nd International Congress on Computer Applications and Computational Science.

[126] Xia C., Lu H., Ooi B.C., Hu J. Gorder: An efficient method for kNN Join processing. Proceedings of the Thirtieth International Conference on Very Large Data Bases.

[127] Wang J., Lin L., Huang T., Wang J., He Z. (2010). Efficient k nearest neighbour join algorithms for high dimensional sparse data. arXiv.

[128] Yu C., Cui B., Wang S., Su J. (2007). Efficient index-based kNN Join processing for high-dimensional data. Inf. Softw. Technol..

[129] Ukey N., Yang Z., Zhang G., Liu B., Li B., Zhang W. Efficient kNN Join over Dynamic High-Dimensional Data. Proceedings of the Australasian Database Conference.

[130] Zhang C., Li F., Jestes J. Efficient parallel kNN Joins for large data in MapReduce. Proceedings of the 15th International Conference on Extending Database Technology.

[131] Garcia V., Nielsen F. Searching high-dimensional neighbours: Cpu-based tailored data-structures versus gpu-based brute-force method. Proceedings of the International Conference on Computer Vision/Computer Graphics Collaboration Techniques and Applications.

[132] Bayer R., McCreight E. (2002). Organization and maintenance of large ordered indexes. Software Pioneers.

[133] Berchtold S., Böhm C., Kriegal H.P. The pyramid-technique: Towards breaking the curse of dimensionality. Proceedings of the 1998 ACM SIGMOD International Conference on Management of Data.

[134] Al Aghbari Z. (2005). Array-index: A plug&search K nearest neighbours method for high-dimensional data. Data Knowl. Eng..

[135] Cayton L. Fast nearest neighbour retrieval for bregman divergences. Proceedings of the 25th International Conference on Machine Learning.

[136] Berchtold S., Keim D.A., Kriegel H.P. The X-tree: An index structure for high-dimensional data. Proceedings of the Very Large Data-Bases.

[137] Lin K.I., Jagadish H.V., Faloutsos C. (1994). The TV-tree: An index structure for high-dimensional data. VLDB J..

[138] Sellis T., Roussopoulos N., Faloutsos C. The R+-Tree: A Dynamic Index for Multi-Dimensional Objects. Proceedings of the 13th International Conference on Very Large Data Bases.

[139] Samet H. (1984). The quadtree and related hierarchical data structures. ACM Comput. Surv. (CSUR).

[140] Eldawy A., Mokbel M.F. Spatialhadoop: A mapreduce framework for spatial data. Proceedings of the 2015 IEEE 31st International Conference on Data Engineering.

[141] Jolliffe I.T. (1990). Principal component analysis: A beginner’s guide—I. Introduction and application. Weather.

[142] Jin H., Ooi B.C., Shen H.T., Yu C., Zhou A.Y. An adaptive and efficient dimensionality reduction algorithm for high-dimensional indexing. Proceedings of the Proceedings 19th International Conference on Data Engineering (Cat. No. 03CH37405).

[143] Mu Y., Yan S. Non-metric locality-sensitive hashing. Proceedings of the AAAI Conference on Artificial Intelligence.

[144] Zhang Z., Ooi B.C., Parthasarathy S., Tung A.K. (2009). Similarity search on bregman divergence: Towards non-metric indexing. Proc. VLDB Endow..

[145] Puzicha J., Buhmann J.M., Rubner Y., Tomasi C. Empirical evaluation of dissimilarity measures for color and texture. Proceedings of the Seventh IEEE International Conference on Computer Vision.

[146] Perronnin F., Liu Y., Renders J.M. A family of contextual measures of similarity between distributions with application to image retrieval. Proceedings of the 2009 IEEE Conference on Computer Vision and Pattern Recognition.

[147] Rasiwasia N., Moreno P.J., Vasconcelos N. (2007). Bridging the gap: Query by semantic example. IEEE Trans. Multimed..

[148] Gray R., Buzo A., Gray A., Matsuyama Y. (1980). Distortion measures for speech processing. IEEE Trans. Acoust. Speech, Signal Process..

[149] Vial P.H., Magron P., Oberlin T., Févotte C. (2021). Phase retrieval with Bregman divergences and application to audio signal recovery. IEEE J. Sel. Top. Signal Process..

[150] Kuang Q., Zhao L. A practical GPU based kNN algorithm. Proceedings of the 2009 International Symposium on Computer Science and Computational Technology (ISCSCI 2009).

[151] Al Aghbari Z., Al-Hamadi A. (2011). Efficient kNN Search by linear projection of image clusters. Int. J. Intell. Syst..

[152] Wang X. A fast exact k nearest neighbours algorithm for high dimensional search using k means clustering and triangle inequality. Proceedings of the 2011 International Joint Conference on Neural Networks.

[153] Satish N., Harris M., Garland M. Designing efficient sorting algorithms for manycore GPUs. Proceedings of the 2009 IEEE International Symposium on Parallel & Distributed Processing.

[154] Chang D., Jones N.A., Li D., Ouyang M., Ragade R.K. Compute pairwise Euclidean distances of data points with GPUs. Proceedings of the iASTED international Symposium on Computational Biology and Bioinformatics.

[155] Kohonen T. (1990). The self-organizing map. Proc. IEEE.

[156] Almalawi A.M., Fahad A., Tari Z., Cheema M.A., Khalil I. (2015). *k* NNVWC: An Efficient *k*-Nearest Neighbours Approach Based on Various-Widths Clustering. IEEE Trans. Knowl. Data Eng..

[157] Zhang J., Zhou X., Wang W., Shi B., Pei J. Using high dimensional indexes to support relevance feedback based interactive images retrieval. Proceedings of the 32nd International Conference on Very Large Data Bases.

[158] Shen H.T., Ooi B.C., Zhou X. Towards effective indexing for very large video sequence database. Proceedings of the 2005 ACM SIGMOD International Conference on Management of Data.

[159] Ilarri S., Mena E., Illarramendi A. (2006). Location-dependent queries in mobile contexts: Distributed processing using mobile agents. IEEE Trans. Mob. Comput..

[160] Doulkeridis C., Vlachou A., Kotidis Y., Vazirgiannis M. Peer-to-peer similarity search in metric spaces. Proceedings of the 33rd International Conference on Very Large Data Bases.

[161] Qu L., Chen Y., Yang X. iDistance based interactive visual surveillance retrieval algorithm. Proceedings of the 2008 International Conference on Intelligent Computation Technology and Automation (ICICTA).

[162] Schuh M.A., Wylie T., Angryk R.A. Mitigating the curse of dimensionality for exact knn retrieval. Proceedings of the Twenty-Seventh International Flairs Conference.

[163] Schuh M.A., Wylie T., Angryk R.A. Improving the performance of high-dimensional knn retrieval through localized dataspace segmentation and hybrid indexing. Proceedings of the East European Conference on Advances in Databases and Information Systems.

[164] Wylie T., Schuh M.A., Sheppard J.W., Angryk R.A. Cluster analysis for optimal indexing. Proceedings of the Twenty-Sixth International FLAIRS Conference, St. Pete Beach.

[165] Boytsov L., Naidan B. (2013). Learning to prune in metric and non-metric spaces. Adv. Neural Inf. Process. Syst..

[166] Weber R., Blott S. (1997). An Approximation Based Data Structure for Similarity Search.

[167] Batcher K.E. Sorting networks and their applications. Proceedings of the Spring Joint Computer Conference.

[168] Liu B., Lee W.C., Lee D.L. Supporting complex multi-dimensional queries in P2P systems. Proceedings of the 25th IEEE International Conference on Distributed Computing Systems (ICDCS’05).

[169] Li M., Lee W.C., Sivasubramaniam A., Zhao J. (2008). Supporting K nearest neighbours query on high-dimensional data in P2P systems. Front. Comput. Sci. China.

[170] Jagadish H.V., Ooi B.C., Vu Q.H., Zhang R., Zhou A. Vbi-tree: A peer-to-peer framework for supporting multi-dimensional indexing schemes. Proceedings of the 22nd International Conference on Data Engineering (ICDE’06).

[171] Clarke L., Glendinning I., Hempel R. (1994). The MPI message passing interface standard. Programming Environments for Massively Parallel Distributed Systems.

[172] Dagum L., Menon R. (1998). OpenMP: An industry standard API for shared-memory programming. IEEE Comput. Sci. Eng..

[173] Luebke D., Harris M. General-purpose computation on graphics hardware. Proceedings of the Workshop.

[174] Corral A., Manolopoulos Y., Theodoridis Y., Vassilakopoulos M. (2000). Closest pair queries in spatial databases. ACM SIGMOD Rec..

[175] Brinkhoff T., Kriegel H.P., Seeger B. (1993). Efficient processing of spatial joins using R-trees. ACM SIGMOD Rec..

[176] Hjaltason G.R., Samet H. Incremental distance join algorithms for spatial databases. Proceedings of the 1998 ACM SIGMOD International Conference on Management of Data.

[177] Koudas N., Sevcik K.C. (2000). High dimensional similarity joins: Algorithms and performance evaluation. IEEE Trans. Knowl. Data Eng..

[178] Böhm C., Braunmüller B., Krebs F., Kriegel H.P. (2001). Epsilon grid order: An algorithm for the similarity join on massive high-dimensional data. ACM SIGMOD Rec..

[179] Kahveci T., Lang C.A., Singh A.K. Joining massive high-dimensional datasets. Proceedings of the Proceedings 19th International Conference on Data Engineering (Cat. No. 03CH37405).

[180] Shim K., Srikant R., Agrawal R. (2002). High-dimensional similarity joins. IEEE Trans. Knowl. Data Eng..

[181] Corral A., D’Ermiliis A., Manolopoulos Y., Vassilakopoulos M. VA-files vs R*-trees in distance join queries. Proceedings of the East European Conference on Advances in Databases and Information Systems.

[182] Achlioptas D. Database-friendly random projections. Proceedings of the twentieth ACM SIGMOD-SIGACT-SIGART Symposium on Principles of Database Systems.

[183] Nálepa F., Batko M., Zezula P. Continuous Time-Dependent kNN Join by Binary Sketches. Proceedings of the 22nd International Database Engineering &Applications Symposium.

[184] Dean J., Ghemawat S. (2008). MapReduce: Simplified data processing on large clusters. Commun. ACM.

[185] Selma C., Bril El Haouzi H., Thomas P., Gaudreault J., Morin M. (2018). An iterative closest point method for measuring the level of similarity of 3D log scans in wood industry. Service Orientation in Holonic and Multi-Agent Manufacturing, Proceedings of the 7th International Workshop on Service Orientation in Holonic and Multi-Agent Manufacturing (SOHOMA’17), Nantes, France, 19–20 October 2017.

[186] Chabanet S., Thomas P., El-Haouzi H.B., Morin M., Gaudreault J. (2021). A knn approach based on icp metrics for 3d scans matching: An application to the sawing process. IFAC-PapersOnLine.

[187] Sakurai Y., Yoshikawa M., Uemura S., Kojima H. (2000). The A-tree: An index structure for high-dimensional spaces using relative approximation. Proc. VLDB.

[188] Ooi B.C., Tan K.L., Yu C., Bressan S. Indexing the edges—A simple and yet efficient approach to high-dimensional indexing. Proceedings of the Nineteenth ACM SIGMOD-SIGACT-SIGART Symposium on Principles of Database Systems.

[189] Arya S., Mount D.M., Netanyahu N.S., Silverman R., Wu A.Y. (1998). An optimal algorithm for approximate nearest neighbour searching fixed dimensions. J. ACM (JACM).

